# Diverse synaptic plasticity mechanisms orchestrated to form and retrieve memories in spiking neural networks

**DOI:** 10.1038/ncomms7922

**Published:** 2015-04-21

**Authors:** Friedemann Zenke, Everton J. Agnes, Wulfram Gerstner

**Affiliations:** 1 School of Computer and Communication Sciences and School of Life Sciences, Brain-Mind Institute, École Polytechnique Fédérale de Lausanne, Lausanne EPFL 1015, Switzerland; 2 Instituto de Física, Universidade Federal do Rio Grande do Sul, Caixa Postal 15051, Porto Alegre RS 91501-970, Brazil

## Abstract

Synaptic plasticity, the putative basis of learning and memory formation, manifests in various forms and across different timescales. Here we show that the interaction of Hebbian homosynaptic plasticity with rapid non-Hebbian heterosynaptic plasticity is, when complemented with slower homeostatic changes and consolidation, sufficient for assembly formation and memory recall in a spiking recurrent network model of excitatory and inhibitory neurons. In the model, assemblies were formed during repeated sensory stimulation and characterized by strong recurrent excitatory connections. Even days after formation, and despite ongoing network activity and synaptic plasticity, memories could be recalled through selective delay activity following the brief stimulation of a subset of assembly neurons. Blocking any component of plasticity prevented stable functioning as a memory network. Our modelling results suggest that the diversity of plasticity phenomena in the brain is orchestrated towards achieving common functional goals.

## Abstract (web summary)

The brain exhibits a diversity of plasticity mechanisms across different timecales that constitute the putative basis for learning and memory. Here, the authors demonstrate how these different plasticity mechanisms are orchestrated to support the formation of robust and stable neural cell assemblies.

## Graphical abstract

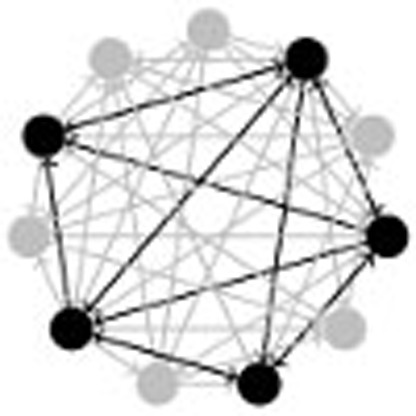

The concepts of cell assembly and Hebbian learning^1^ have inspired generations of experimental and theoretical work^2^. A cell assembly, loosely formulated as a group of neurons with strong connections among each other, can be interpreted as a functional circuit of brain activity. Cell assemblies may be activated during memory recall, as evidenced by delay activity of neurons during working memory tasks^3^,^4^ or during recognition of abstract items^5^,^6^,^7^. While models of cell assemblies for fixed, preset connectivity can be readily constructed^8^,^9^,^10^,^11^, the question of whether Hebbian learning rules can be used to *form* and *recall* such assemblies in a robust, stable manner is not well understood^12^,^13^,^14^.

The reason why models fail to form functional memory assemblies in plastic networks of spiking neurons could be linked to either one specific or a combination of several features of biological networks, which were not addressed in these models. First, there are many different types of neurons in the brain, and experimental forms of plasticity depend on both the type of neuron and its connections^15^. Second, plasticity manifests in multiple concurrently active forms. This includes, but is not limited to rate-dependent^16^, voltage-dependent^17^ and spike-timing-dependent^18^,^19^,^20^ homosynaptic as well as heterosynaptic plasticity^21^,^22^. Third, induction of synaptic plasticity needs to be distinguished from processes of synaptic consolidation and maintenance^23^,^24^. Finally, additional nonstandard forms of plasticity such as structural plasticity^25^,^26^, short-term plasticity (STP)^27^,^28^ or homeostatic synaptic changes^29^ complicate the picture.

Here we show that a well-orchestrated combination of a plausible Hebbian plasticity model^30^ together with non-Hebbian forms of plasticity and globally modulated inhibitory plasticity leads to the formation of cell assemblies. Importantly, the emergent assemblies are stable and do not degrade or inflate during ongoing activity and memory recall. In order to distinguish different forms of plasticity in our model, we use the following terms and criteria. First, we call contributions to synaptic plasticity that depend only on the state of the postsynaptic neuron, but not on those of the presynaptic neurons, ‘heterosynaptic'. Manifestations of synaptic plasticity that depend jointly on pre- and post-synaptic activity are called ‘homosynaptic'. Similarly, changes of the synapse that depend only on the transmitter release, but not on the state of the post-synaptic neuron, are called ‘transmitter-induced'. By definition, heterosynaptic and transmitter-induced plasticity are non-Hebbian, while homosynaptic plasticity can either be Hebbian or anti-Hebbian. Second, in our terminology we also consider the timescale on which synaptic changes manifest themselves. We refer to slow compensatory processes that act on a timescale above 10 min as ‘homeostatic'. They are contrasted by rapidly ‘induced' plasticity caused on the one hand by typical plasticity protocols (lasting a few seconds to tens of seconds), for example, for the induction of long-term potentiation (LTP) and depression (LTD), and on the other hand by fast compensatory mechanisms that include non-Hebbian forms of plasticity. Third, a mathematical rule of synaptic plasticity is considered as ‘local' if it depends only on the activity of the presynaptic neuron and the state of both synapse and postsynaptic neuron. Moreover, a plasticity rule can be under the influence of global factors^31^,^32^,^33^ such as neuromodulators^33^,^34^ or other secreted factors^35^. Note that in this nomenclature ‘local' does not exclude ‘heterosynaptic'^21^. We show that the concerted action of local homosynaptic, heterosynaptic and transmitter-triggered forms of plasticity at excitatory synapses leads to stable assembly formation and recall in recurrent networks of spiking neurons.

## Results

**Figure 1:**
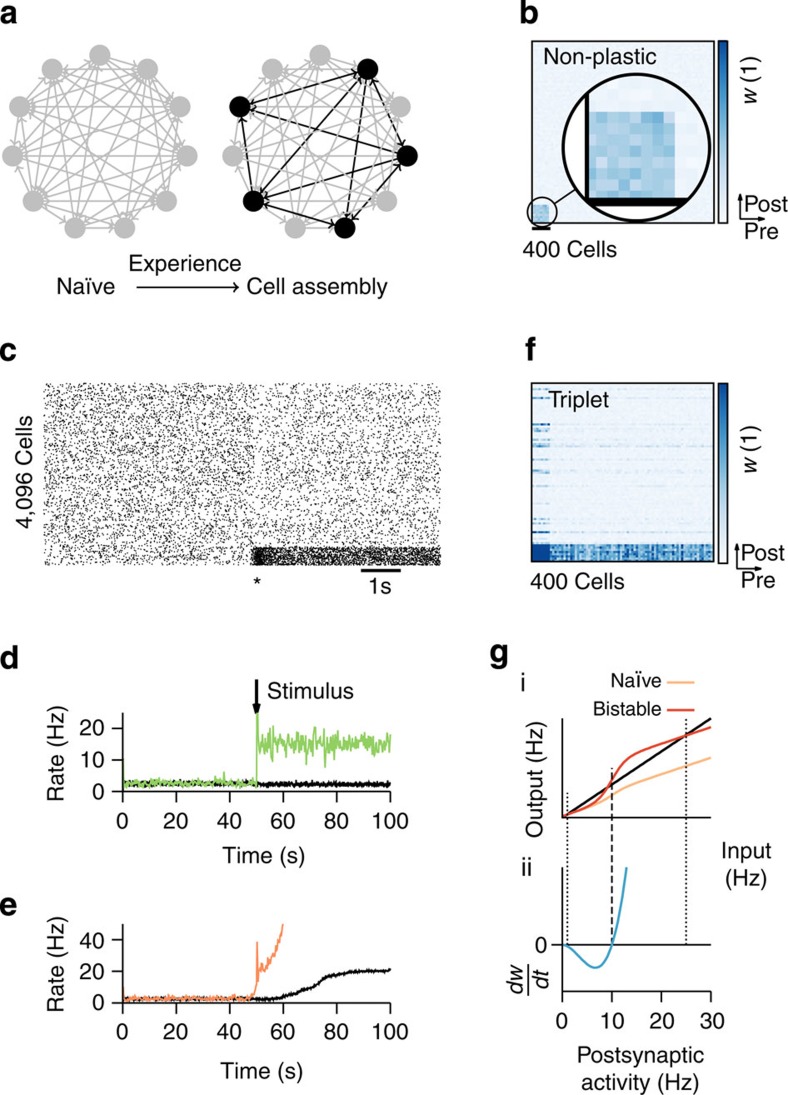
Classical synaptic learning rules fail to maintain stable cell assemblies in recurrent neural networks. (**a**) Schematic network with random sparse connectivity before any experience (naïve; left) and with an embedded cell assembly (right). (**b**) Two-dimensional (2D) histogram of the excitatory recurrent synaptic weights. Synaptic weights within the cell assembly (zoom) are preset to be stronger (darker shading) than other weights in the network (light tone). (**c**) Spike raster from a recurrent network model with a single embedded cell assembly. At * a 300-ms external stimulation is applied to the cells within the assembly which triggers persistent delay activity. (**d**) Population rate of cells outside of the cell assembly (black) and inside the assembly (green). (**e**) Same as in **d**, but for a plastic network with triplet STDP^30^. Activity inside the cell assembly (red) explodes rapidly and the activity of the other cells (black) follows. (**f**) Same as **b**, but at the end of 100 s of simulation of the plastic network. Many connections into the assembly have been potentiated. (**g**,**i**) Schematic representation of the graphical solution of a nonlinear system describing the firing rate dynamics within a neural population before and following learning. Stationary solutions can be found graphically as the intersection of the diagonal (black) and the effective input–output curve of neurons within the population (red). Before the encoding of a cell assembly (naive, orange line) there exists only one solution at low firing rates. Synaptic potentiation can alter the response such that three solutions exist (red line; see Supplementary Fig. 1a–d). Stable stationary solutions are marked by dotted vertical lines. (ii) Synaptic changes (
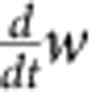
, vertical) as a function of the rate of the postsynaptic neuron in Bienenstock–Cooper–Munro or triplet STDP (blue). At low (high) postsynaptic activity synaptic long-term depression (potentiation) dominates. The plasticity rule has no stable fixed point at ≈25 Hz corresponding to the stable high-rate fixed point of the network dynamics (i). Therefore, if the postsynaptic neuron fires at 25 Hz, all weights continue to increase.

We simulated a network of 4,096 excitatory and 1,024 inhibitory randomly connected integrate-and-fire neurons containing a cell assembly of 400 excitatory neurons. In a first experiment, the assembly is defined by intra-assembly synapses that are initialized at stronger values than those of the rest of the network (Fig. 1a,b). In the absence of plasticity, the network functions as a working memory that exhibits delay activity (Fig. 1c,d) consistent with earlier findings^9^,^10^,^11^,^13^,^36^; however, when we switch on a standard homosynaptic model of spike-timing-dependent plasticity^30^ (STDP), the activity of neurons within the assembly, characterized by their firing rates, increases dramatically, followed by a slower increase in neuronal activity outside the assembly (Fig. 1e).

### Network dynamics interact with plasticity

The biologically unrealistic increase in firing rates in this and similar, homosynaptic Hebbian models^37^,^38^,^39^ results from an interaction of the network dynamics with synaptic plasticity^13^. The change of synaptic weight from a presynaptic neuron *j* to a postsynaptic neuron *i* in standard homosynaptic plasticity models such as the classical Bienenstock–Cooper–Munro rule^40^ or modern N-methyl-D-aspartate (NMDA) receptor-dependent^39^, spike-timing-dependent^30^,^38^ or voltage-dependent^41^ variants requires that the activity (pre)_*j*_ of the presynaptic neuron is multiplied with the activation of some postsynaptic variables (post)_*i*_ that can be summarized as Δ*w*_*ij*_∝(pre)_*j*_ × (post)_*i*_ × *F*((post)_*i*_–*θ*_*i*_), with a function *F* that vanishes if (post)_*i*_=*θ*_*i*_ (for example, *F*(*x*)=*x*; *θ*_*i*_ is the threshold for LTP). It is a homosynaptic rule because a synapse that is not presynaptically activated ((pre)_*j*_=0) does not change. If presynaptic activity occurs (pre)_*j*_ >0 then it depends on the present state of the postsynaptic neuron whether the weight changes upwards or downwards. Even in the presence of presynaptic activity, the weight change is zero if the postsynaptic variable is zero (post)_*i*_=0 or equal to the threshold *θ*_*i*_. Activity values at which the weight does not change are called fixed points of the synaptic dynamics.

The synaptic dynamics interact with the neuronal dynamics of cell assemblies in a memory network. During memory recall, the assembly is strongly active while background neurons (that is, those not participating in the assembly) show weak spontaneous activity (Fig. 1c). In the presence of a homosynaptic plasticity rule with the above structure, the spontaneous activity of background neurons and the higher activity of the assembly neurons leads to an increase in all those synapses projecting on an assembly neuron *i* (Fig. 1f). This finding can be understood in the framework of graphical network analysis of working memory models^11^. During memory recall, assembly neurons receive input from neurons of the same assembly. Input firing rates are transformed into output firing rates by an effective transfer function, closely related, but not identical, to the neuronal f–I curve. Stable memory recall requires that the rates of input neurons (that is, cells in the assembly) and output neurons (other cells in the same assembly) match (Fig. 1gi). Since memory recall should not change the contents of the memory, the overall dynamics during recall has to be at a fixed point. However, in general, there is a mismatch between the network dynamics and synaptic dynamics (Fig. 1gii). Matching the fixed points by a compensatory shift of the threshold *θ*_*i*_ (ref. 40) only succeeds if the shift is faster than the dynamics of induced synaptic plasticity^42^. It is therefore inconsistent with the notion of homeostasis as a slow adaptation towards a physiologically desired state.

### Orchestrated plasticity

We wondered whether a combination of different forms of plasticity could work in concert to match the fixed points of network and synaptic dynamics. To do so, we orchestrated several distinct forms of plasticity in a single model. This model comprises STP, homosynaptic LTP and LTD, heterosynaptic up- and downregulation of synapses and transmitter-induced plasticity as well as consolidation.

STP is the fastest form of plasticity in our model. Like in earlier models^27^,^28^, short-term depression contributes to a robust firing rate bistability in a cell assembly because the saturation of synapses at high presynaptic frequencies provides the effective transfer function with the curvature^43^,^44^,^45^ necessary for a stable fixed point at intermediate firing rates (Supplementary Fig. 1a–d and Supplementary Methods). Thus, short-term depression counteracts the linearization of f–I curves observed with adapting neurons^46^,^47^.

### Orchestrated plasticity makes synapses bistable

**Figure 2:**
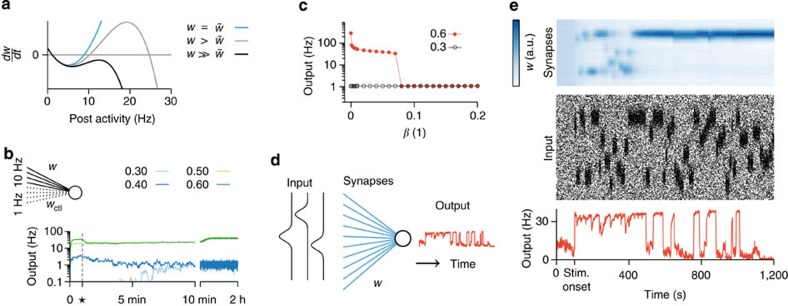
Orchestrated forms of plasticity in a feed-forward network give rise to bistability and synaptic competition. (**a**) Postsynaptic rate dependence of orchestrated forms of plasticity. With a synaptic weight larger than the reference value 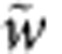, synaptic depression is possible at high postsynaptic rates. The blue curve 
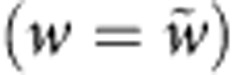
 is the same as in Fig. 1g,ii. (**b**) The mean firing rate over time of a single postsynaptic integrate-and-fire neuron with plastic synapses for different initial conditions. The postsynaptic neuron receives simultaneous Poisson input via an active pathway (80 synapses at 10 Hz) and a control pathway (80 synapses at 1 Hz, initial weight *w*_ctl_=0.1; see schematic at top left; *cf.* Fig. 3). Different colours signify different initial weights *w* in the active pathway. Plasticity is activated after 1 min of burn-in time (*, dashed line). Inset: zoom on different initial firing rates at plasticity onset. Note the change in timescale after 10 min. Synaptic weights of the control pathway: Supplementary Fig. 3c,d. (**c**) The mean firing rate of the same neuron as in **b** after 2 h of simulated time for different values of the parameter *β* of heterosynaptic plasticity. Black (red) data points are from simulations with initially lower (higher) synaptic afferent weights. (**d**) Schematic of the simulation of a single integrate-and-fire neuron receiving spatiotemporally correlated input. (**e**) Simulation results with the paradigm in **d**. Top panel: evolution of the synaptic weights indicates receptive field development. Middle panel: spike raster of the input spike trains. Bottom panel: The mean firing rate of the postsynaptic neuron over time. After stimulus onset (at *t*=100 s) a Gaussian activity profile centred at a random position modulates the input spike trains. The centre of the Gaussian is shifted at random intervals (mean=20 s).

The formation of cell assemblies proceeds via Hebbian plasticity at excitatory synapses^1^. In our model we use a standard triplet STDP rule of long-term plasticity^30^, which is, like all forms of Hebbian plasticity, unstable^48^. Stability is restored through the direct interaction of triplet STDP with two local non-Hebbian plasticity mechanisms that act on the same timescale. First, at low rates, transmitter-induced potentiation counteracts homosynaptic LTD in a push–pull manner to prevent the network from falling silent. Similarly, at high rates, heterosynaptic depression prevents explosive run-away potentiation of strong synapses. For sensible parameter choices the interplay of the three forms of plasticity generates two stable fixed points of the weight dynamics, which coincide with the activity fixed points of neurons in cell assemblies (Fig. 2a)—one at low firing rates (≈1 Hz) and one at elevated firing rates (≈30 Hz; Supplementary Figs 1–3).

To check for bistability in simulations, we stimulated a single postsynaptic neuron with Poisson spike input from 80 presynaptic excitatory neurons at 10 Hz, while 80 other presynaptic excitatory neurons firing at 1 Hz served as a control. Depending on the strength of synaptic weights at the beginning, the firing rate converged during the simulation to one of two different values (Fig. 2b; Supplementary Fig. 3). In all cases firing rates at first evolved quickly through the action of rapidly induced forms of plasticity (Fig. 2b), which was followed by slow changes on the timescale of consolidation dynamics (Methods; Fig. 2b; Supplementary Fig. 3b,d). Importantly, synaptic weights did not saturate at the maximally allowed value (*w*^max^=5), but stayed at intermediate levels whose values were pathway-dependent (Supplementary Fig. 3c,d). The firing rate at the upper stable state was dependent on the parameter *β* that characterizes the strength of heterosynaptic plasticity (Fig. 2c; Methods) and on the number of synaptic inputs (Supplementary Fig. 3g,h), while the firing rate in the other stable state depended on the parameter *δ* of the transmitter-induced form of plasticity (Supplementary Fig. 1f) and was independent of the number of connections (Supplementary Fig. 3g). When stimulated with a localized stimulus that changes its position on average once every 20 s, the postsynaptic neuron develops a localized receptive field (Fig. 2d,e), similar to earlier models^40^,^41^,^49^.

In a recurrent network, the fixed point of synaptic plasticity at elevated firing rates is, based on theoretical arguments (*cf.* Figs 1g and 2a), expected to self-adjust to match the dynamics of a cell assembly, provided the network activity remains asynchronous and the number of neurons partaking in each cell assembly is limited. Like in previous models^10^,^11^ the latter is achieved through global inhibition. In our model, inhibitory synapses are subject to a spike-timing-dependent learning rule which is reminiscent of experimental results^50^ and modulated by a hypothetical global secreted factor (Methods). This modulation allows inhibitory plasticity to regulate activity globally^35^ at the network level during ongoing plasticity at excitatory synapses.

### Assembly formation and recall

**Figure 3:**
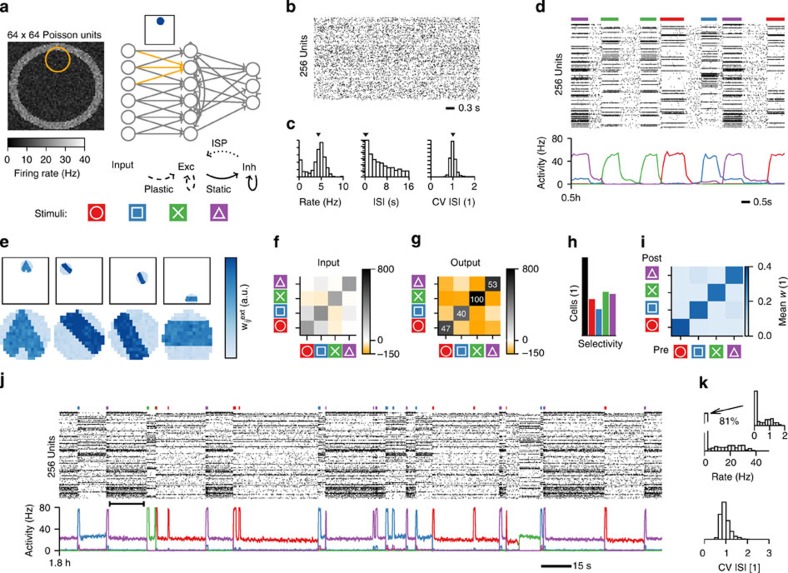
Stable formation and recall of memory assemblies. (**a**) Schematic representation of the network model (top right). Top left: input layer composed of 64 × 64 Poisson neurons. Each network neuron receives input from a circular subset (orange circle) of input units centred at a random position. Inset: initial receptive field of a network neuron (compare **e**). Connection types are indicated below. Solid lines: no long-term plasticity. Dashed lines: orchestrated Hebbian and non-Hebbian plasticity. Dotted line: inhibitory plasticity (ISP). Four overlapping geometric shapes (bottom) are presented at random times via the input units. (**b**) Spike raster of the initial network activity. (**c**) Network statistics from **b**: histograms of the firing rates, the interspike intervals (vertical axis logarithmic) and the coefficient of variation of the interspike intervals (CV ISI). The mean values indicated by arrow heads. (**d**) Network activity after 30 min. Top: spike raster. Coloured bars indicate time, duration and identity of the stimuli (*cf.* **a**). Bottom: temporal evolution of the population firing rate of assembly neurons coding for the respective stimuli (Methods). (**e**) Receptive fields of four randomly chosen network neurons after learning. Points represent existing connections and their position in the 2D input space (compare **a**). Colour encodes the connection strength. Bottom: zoom on receptive fields. The initial state of panel 1 is shown in **a**. (**f**) Covariance matrix of stimulus-evoked firing rates of the input neurons (Methods). (**g**) Same as **f**, but for the observed network activity after learning. Numbers on the diagonal indicate the percentage of the maximum value. (**h**) Bar plot illustrating the relative fraction of cells selective per stimulus. Black: no preference. Colours as in **a**. (**i**) The mean weight strength of the recurrent weights between neurons ordered according to stimulus preference (*t*=1 h). (**j**) Same as **d**, after ∼1.8 h of simulated time. Note the delay activity during the interstimulation interval (after *t*=1 h: *T*_Off_=20 s). For clarity only every fifth spike is plotted. Black range bar indicates data range used for spike statistics in **k**. (**k**) Histograms of firing rates and CV ISI in the network during the interval marked in **j**.

**Figure 4:**
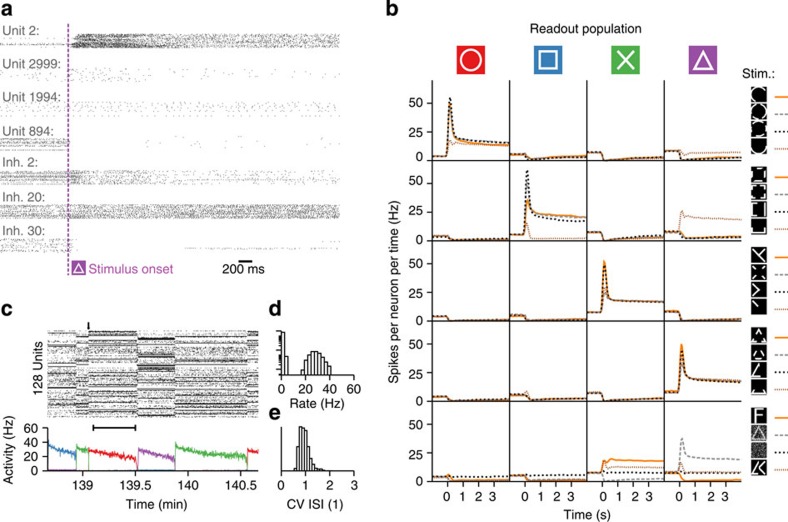
Network states are associative and serve as working memory. (**a**) Spike raster of typical single unit responses to a particular stimulus. From top to bottom: four excitatory units (one assembly neuron coding for ‘triangle', two background units and one neuron coding for a different assembly) as well as three randomly selected inhibitory neurons are shown. The different trials (*n*=30) are aligned on stimulus onset (dashed line). (**b**) Population-averaged peristimulus time histograms (PSTH) of the four relevant readout populations for all different distorted stimuli presented (keys on the right). (**c**) Spike raster of 128 excitatory neurons with a slow spike-triggered adaptation current^54^ with a time constant of 20 s (*cf.* Methods). At around 139 min the currently active assembly is switched off by a brief external stimulus (arrow). All other state changes are spontaneous. (**d**) Histogram of firing rates averaged over the interval marked with a black bar in **c**. Vertical axis logarithmic. (**e**) Histogram of coefficient of variation of the interspike interval distribution (CV ISI) averaged over the interval marked in **c**.

We studied whether the combination of inhibitory plasticity with excitatory homosynaptic, heterosynaptic and transmitter-induced plasticity could work in symphony to enable stable assembly formation and recall in a spiking recurrent network model. To that end we implemented all forms of plasticity described above in the random network of excitatory and inhibitory neurons (Fig. 3a). Each excitatory neuron received recurrent input from the network, but also from a small patch of sensory neurons that defines the spatial location of its receptive field (Fig. 3a). All excitatory synapses were initialized with a common value such that the recurrent network exhibited asynchronous irregular firing^51^,^52^ (Fig. 3b,c). Synapses evolved freely according to the orchestrated plasticity rules described above. The network was then stimulated by applying repeatedly and stochastically one of four possible full-field input patterns (Fig. 3a,d). Stimulus identity, stimulus duration and interstimulus interval were randomized (Methods), while the stimulus intensity was kept fixed. Plasticity of feed-forward synapses induced the development of spatially structured feature detectors within the receptive fields (Fig. 3e) that caused neurons to respond to specific input patterns (Fig. 3g,h). Plasticity of recurrent excitatory connections led to the development of strongly connected assemblies (Fig. 3i), reminiscent of recent experimental findings in the sensory cortex^53^. In our model, however, recurrent connections grew strong enough that assemblies could sustain selective delay activity following a brief stimulation of one of the patterns (Fig. 3j; Supplementary Movie 1) consistent with signatures of attractor dynamics in experiments^3^,^4^,^5^,^6^,^7^. Neurons that participated in an assembly exhibited a broad range of firing rates during delay activity (Fig. 3k). Background neurons had a firing rate of around 1 Hz or less (Fig. 3k) and showed a large trial-to-trial variability (Fig. 4a). Some background neurons exhibited weakly inhibited or elevated responses to a specific assembly, whereas others did not (Fig. 4a). A large fraction of neurons did not belong to any of the assemblies (that is, never fired at high rate; Fig. 3h), which suggests that there is a ‘reserve pool' that could become sensitive to novel patterns not included in the stimulation paradigm (see below).

To check whether recall is associative, we stimulated the plastic network with partial input by occluding up to three quarters of the input field. In most cases, we found activation of the appropriate assembly corresponding to the partial information, indicating memory recall from partial cues (Fig. 4b; Supplementary Fig. 4a). Despite ongoing plasticity the learned assemblies were stable and did not degrade during days of ongoing network activity (Supplementary Fig. 4b). Completely novel stimuli, unrelated to those previously encountered, or an ambiguous combination of known patterns could initiate memory recall of a single memory with overlap with the stimulated pattern (Fig. 4b; Supplementary Fig. 4a). In some rare cases memory recall failed and could lead to a brief deactivation of all assemblies. However, this background state was generally short-lived and followed by the spontaneous activation of one of the stored assemblies. Without external input, spontaneous state transitions occurred occasionally after several minutes of selective delay activity of a single pattern. On shorter timescales the individual neuronal firing rates during delay activity were approximately stable. However, the inclusion of neuronal adaptation on a timescale of seconds^54^ (Methods) caused firing rates to change more rapidly and state transitions between memory items to occur more frequently (Fig. 4c–e; Methods). Nevertheless, state transitions could still be caused by external stimuli, indicating that the recurrent network remained responsive to sensory input.

### Timescales of plasticity

**Figure 5:**
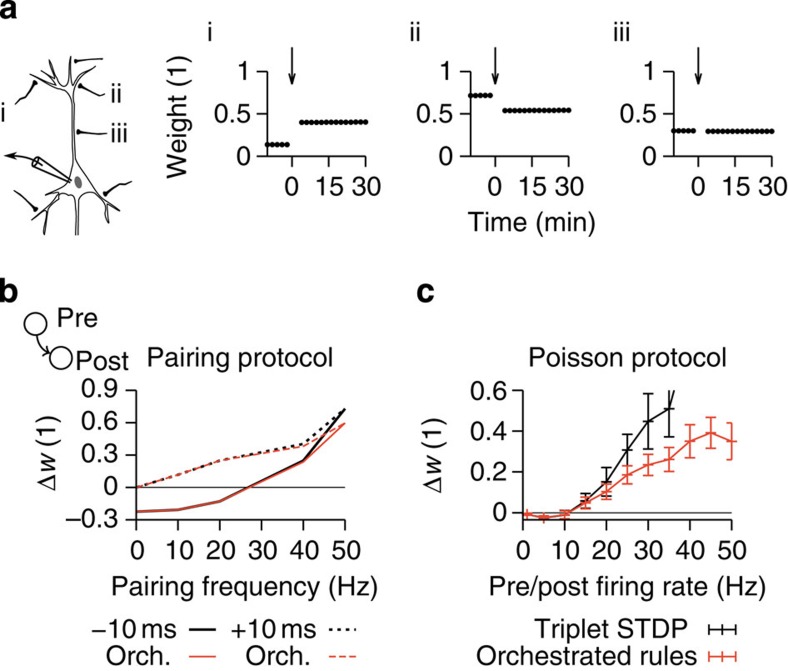
Differential induction of orchestrated forms of plasticity. (**a**) Bidirectional non-Hebbian plasticity in our point neuron model. Plasticity is induced by postsynaptic tetanization as described in ref. 49. At time *t*=0 the postsynaptic neuron is forced to spike (three burst trains at 1/60 Hz, ten bursts per train at 1 Hz, five spikes per burst at 100 Hz). Depending on its initial state a synapse exhibits LTP (i), LTD (ii) or no change at all (iii). (**b**) Relative weight change caused by a classical pairing protocol with pre-before-post (+10 ms; solid lines) and post-before-pre (−10 ms; dashed lines) spike timing at different pairing frequencies^20^ for orchestrated plasticity (red) and minimal triplet STDP model without homeostatic regulation of LTD (black). Below 40 Hz the frequency dependence of STDP does not change for orchestrated plasticity rules compared with triplet STDP. Protocol: 75 simulated pairings at +10 and −10 ms spike timing. (**c**) Relative weight change under a plasticity protocol in which the pre- and postsynaptic cell fire Poisson spike trains with 5-ms absolute refractory period. Classical triplet STDP model (black) is different from orchestrated plasticity (red). The duration of the Poisson spike trains is scaled to keep an expected number of 100 spikes for the pre- and postsynaptic spike train and the same plasticity models as in **b** are used. Error bars indicate the s.d. of *n*=20 independent trials.

The stability in our model is a direct consequence of the orchestrated interplay of multiple plasticity mechanisms on different timescales. First, on the timescale of several hundred milliseconds the nonlinearity of STP creates the possibility for firing rate bistability in cell assemblies at intermediate levels of neuronal activity (see Supplementary Fig. 1b,c; Supplementary Methods). Second, on the timescale of seconds, induction of plasticity is achieved by a combination of triplet STDP with heterosynaptic and transmitter-induced plasticity (see Methods). Transmitter-induced plasticity of strength *δ*, in our model, is proportional to the presynaptic activity (pre)_*j*_ and ensures low neuronal baseline firing rates^55^. Similar to earlier models^21^,^49^, heterosynaptic plasticity of strength *β* changes all synapses on neuron *i* whenever the postsynaptic activity (post)_*i*_ reaches a high value. The direction of change depends on the present value *w*_*ij*_ of the synaptic weight in relation to a reference weight 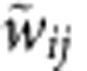 consistent with experiments of tetanic burst induction^49^ (Fig. 5a). The combination of transmitter-induced, heterosynaptic and Hebbian plasticity at the excitatory synapse between neuron *j* and the postsynaptic neuron *i* induces weight change schematically described by


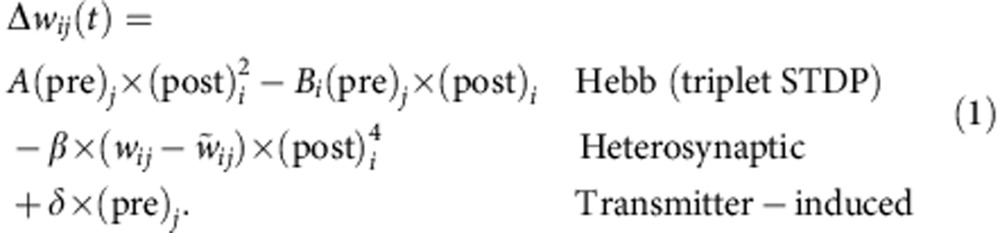


In the triplet STDP model, LTP is quadratic in the postsynaptic variable and of strength *A*, whereas LTD is linear in the postsynaptic variable and of strength *B*_*i*_. The fourth power in the description of heterosynaptic plasticity implements a threshold on the postsynaptic activity and acts as a burst detector (*cf.* Fig. 2a). In standard STDP protocols, the heterosynaptic effect is small and equation (1) describes frequency dependence of STDP^20^,^30^ (Fig. 5b). If, however, pre- and postsynaptic neurons fire stochastically at high rates, the heterosynaptic term counteracts homosynaptic LTP (Fig. 5c; ref. 49). This creates the additional stable fixed point at elevated firing rates, necessary to co-stabilize plasticity and assembly dynamics (Fig. 2a; Supplementary Fig. 2b).

Third, processes such as homeostasis or consolidation that act on timescales much longer than plasticity induction may influence the stability of induced synaptic weight changes. In the simulations of Figs 1, 2, 3, 4, we have neglected homeostasis and preconfigured the network with appropriate parameter values. In particular, we initialized the weight values of the external afferent connections such that novel stimuli evoked responses in network neurons strong enough to drive them above the LTP threshold. A 20% change of the initial weight value in either direction did not yield a qualitatively different outcome of the simulation. However, when weights were initially too weak or afferent connections were chosen randomly (that is, no predefined spatial receptive fields) all neurons remained at the low activity fixed point and no assembly structure was formed (Supplementary Fig. 5a).

This behaviour was changed when we allowed the strength of LTD to change through homeostatic metaplasticity on the timescale of tens of minutes to hours^30^,^40^,^41^,^56^. With homeostatic plasticity our model formed cell assemblies even when the initial weights were weak and connectivity was random (Supplementary Figs 6 and 7). The behaviour of the network was similar to Fig. 3, except that, in addition, we noticed the emergence of a single random pattern that was not correlated to any of the learned stimuli (Supplementary Fig. 7a).

### Learning of novel memories

**Figure 6:**
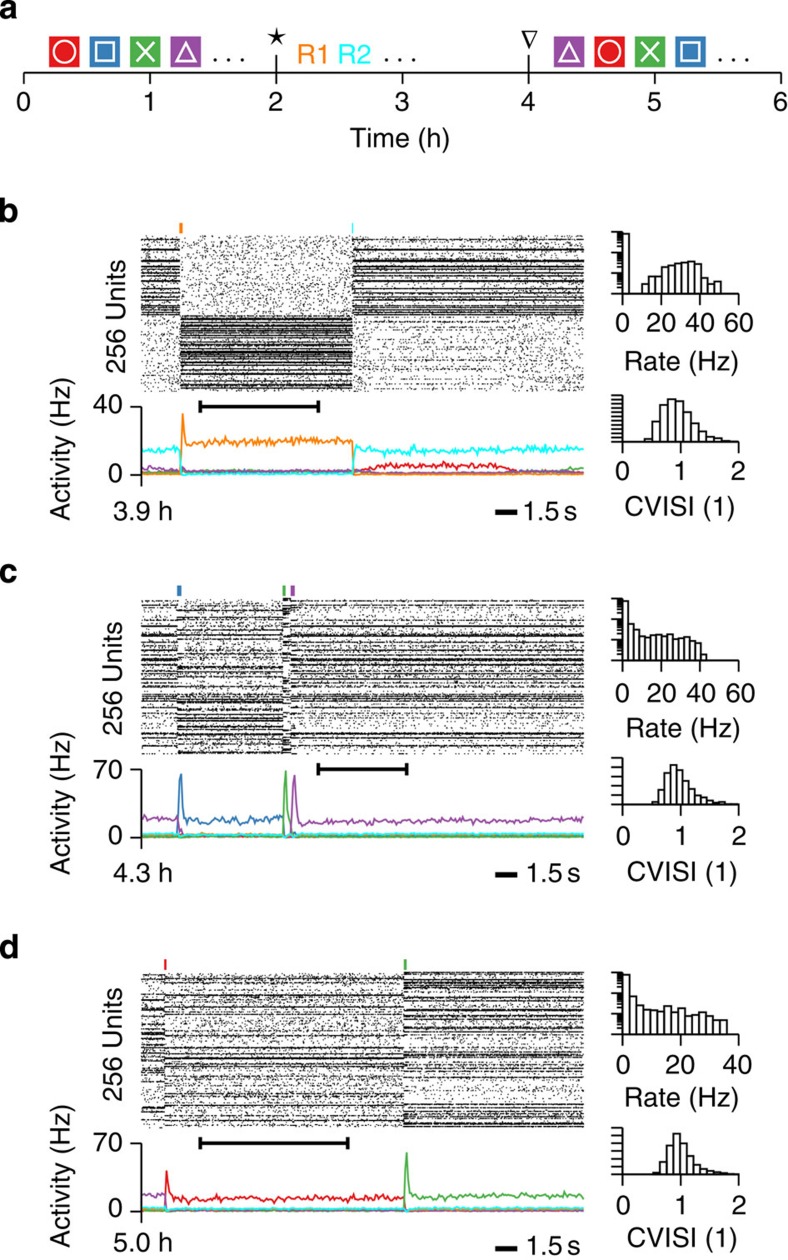
Novel patterns can be stored in addition to old patterns through external activation. (**a**) Illustration of the stimulation protocol. After 2 h of stimulation (*) with the original input modality (*cf.* Fig. 3), two subsets of neurons R1 (yellow) and R2 (cyan) were stimulated repeatedly for 2 h through synapses from a different input modality (Methods). At ∇ the input modality is reverted back to the same as the previous. (**b**) The network activity towards the end of the stimulation phase of R1/R2. Spike raster (top left) and population activity (bottom left) are shown. The histograms on the right characterize the firing rates and the CV ISI of the spiking activity during the interval highlighted with the black bar in the raster plot on the left. (**c**) Same as **b**, but shortly after the original synaptic input was restored (*cf.* ∇ in **a**). (**d**) Same as **c**, but 1 h after the original synaptic input was restored.

Biological networks retain the ability to acquire new memories over time. Since after 2h strong receptive fields had already been formed, novel stimuli, presented within the same visual stimulation paradigm, were classified as one of the four learned patterns and did not lead to novel memories, even if presented repeatedly (Supplementary Fig. 8). To test whether the recurrent connections could in principle store new assemblies, we continued the simulation of the network from Fig. 3, but stimulated two pools (R1/R2) of network neurons through synaptic input from a different input modality (Fig. 6a,b; Methods). As a consequence, the two pools formed new assemblies capable of maintaining selective delay activity (Fig. 6b). During recall of the new assemblies, we noticed the occasional partial activation of one of the previous patterns with one of the new patterns (Fig. 6b), which might be linked to overlap between the assemblies. When input from the original input modality was restored after 2 h, there was no notable effect on the recall behaviour of the four original assemblies (Fig. 6c,d), indicating that new memories can be added without destroying existing ones.

### Stability of memories

**Figure 7:**
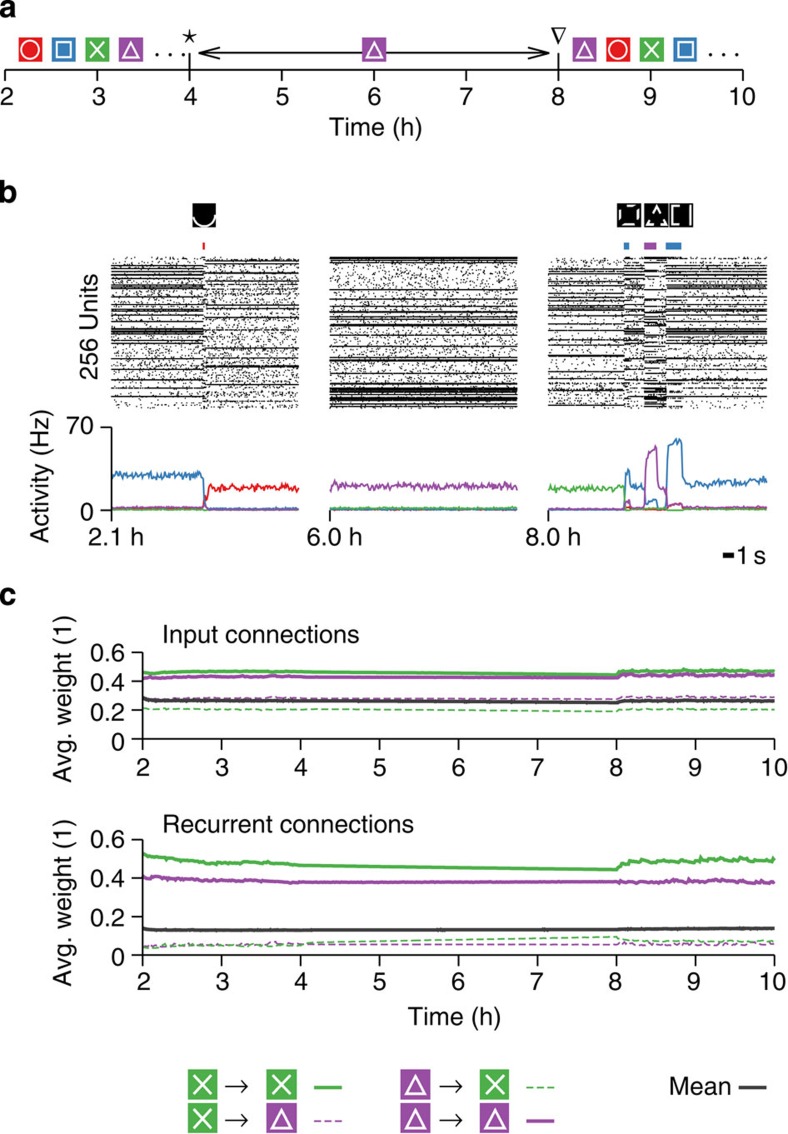
Learned synaptic structures are stable during prolonged recall. (**a**) Schematic overview of the protocol. Continuation of the simulation shown in Fig. 3. Initially the network recalls stored patterns from distorted cues. During the interval from * to ∇ a single pattern (violet) is made continuously active, before external stimulation is resumed. (**b**) Three representative snapshots of network activity during the three phases. Spike raster (middle), stimulus identity and duration (top) and pattern activity (bottom). (**c**) Average weight of representative subsets of input connections (top) and recurrent connections (bottom) during the protocol illustrated in **a**. The overall mean of all synaptic weights is plotted in black in both cases.

Spontaneous or induced memory recall potentially plays a role in refreshing previously stored memories^57^. To test the stability of synaptic weights of an inactive assembly during the extended recall of another pattern, we recorded the evolution of synaptic weights during a 4-h-long activation period of a single assembly (Fig. 7a,b; Methods). During this period external connections to both, active and inactive cell assemblies, as well as recurrent connections of the active assembly, hardly changed (Fig. 7c). Connections within an inactive assembly showed a slow decay. Recall from partial cues was demonstrated 4 h later (Fig. 7b).

### Role of consolidation

**Figure 8:**
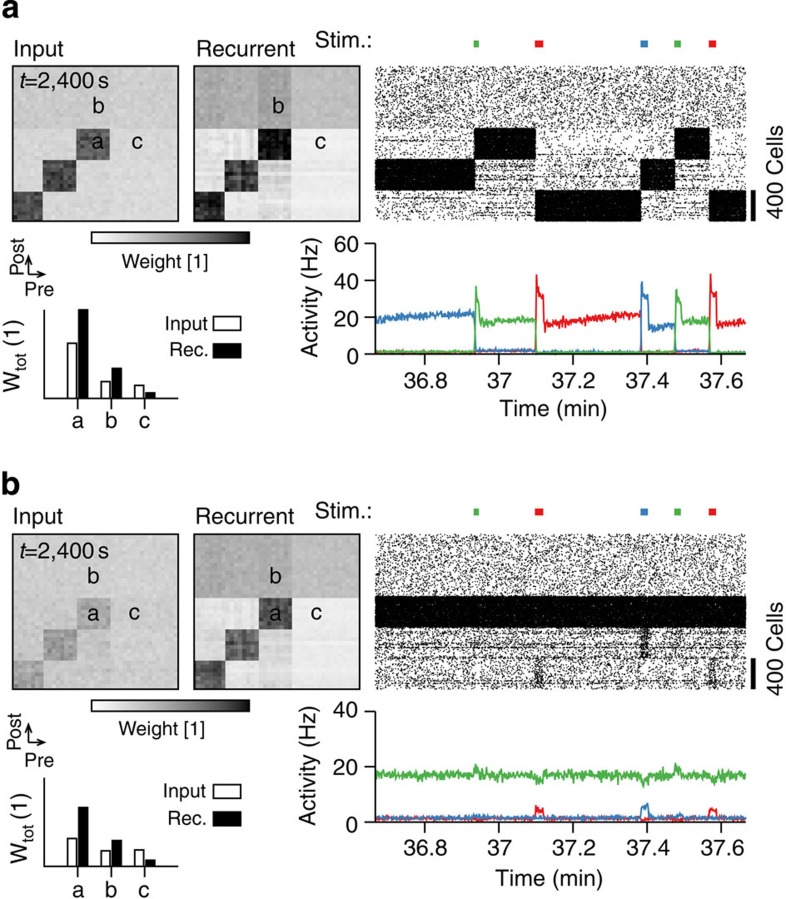
Blocking consolidation causes cell assemblies to decouple from external input. (**a**) Control network with normal consolidation dynamics. Top left: 2D histogram of a 2,000-unit section of the excitatory weight matrix that connects input neurons with the excitatory neurons in the network. Characteristic values have been plotted in the bar plot at the bottom (highlighted letters). Top middle: as before but showing recurrent weights between the selected 2,000 units in the network. Top right: spike raster of the same 2,000 units. Bottom right: population activity of the 3 × 400 unit blocks shown in the spike raster. (**b**) Same as in **a**, but for the network without consolidation. Input weights are weaker (left) and network activity is only marginally affected by input (right). Periods of stimulation indicated by the coloured bars above the spike raster.

In our model, synaptic weights are consolidated^24^ on the timescale of 20–60 min (Supplementary Fig. 2; Methods). To study the effects of the consolidation dynamics, we considered two identical network simulations and blocked the consolidation dynamics in one of them, while the other network served as a control. Both networks were prepared with an initial feed-forward connectivity in which three blocks of input neurons, consisting of 400 neurons each, were connected through strong connections (*w*_*ij*_) to equally sized blocks of neurons in the network (Supplementary Fig. 9a,b). All recurrent connectivity was initially unstructured and all reference weights 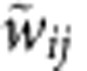 were initialized at zero (Supplementary Fig. 9a,b; Methods). After ∼13 min of repeated stimulation of each of the three inputs, both recurrent networks had formed cell assemblies corresponding to these three inputs and exhibited selective delay activity (Supplementary Fig. 9c,d), while other neurons in the network remained at low firing rates. After 20 min of simulated time we increased the mean interstimulation interval from *T*_Off_=2 s to *T*_Off_=20 s. While external stimulation in the control network continued to reliably evoke switches of the network state during the subsequent 20 min (Fig. 8a; Supplementary Fig. 9e), the network with blocked consolidation showed little response to external stimulation so that delay activity was mostly decoupled from the external input (Fig. 8b; Supplementary Fig. 9f). The effect was accompanied by a decrease in the synaptic strength of the input and the recurrent connections projecting on the respective assemblies in the consolidation-blocked (Supplementary Fig. 9d,f), but not in the control network (Supplementary Fig. 9c,e). Thus, a network without synaptic consolidation can no longer recall memories from external cues. These findings suggest a potentially important computational role of consolidation.

### Blockage of individual plasticity mechanisms

To further investigate the individual roles of the various plasticity mechanisms in our model we ran additional simulations in which specific forms of plasticity were selectively disabled. First, when disabling transmitter-induced plasticity the network was able to form and recall cell assemblies. However, about one third of all cells in the network became quiescent during the induction protocol and remained silent thereafter (Supplementary Fig. 5b). The deactivation of heterosynaptic plasticity alone (*β*=0) resulted in an immediate and irreversible increase in neuronal firing rates following stimulation that prevented the network from forming useful stimulus representations or working memory states (Supplementary Fig. 5c).

Finally, blocking inhibitory plasticity at the beginning of the simulation resulted in substantially higher firing rates, although it did not prevent the network from learning or function as a working memory (Supplementary Fig. 5d). However, when we first simulated the full network with all plasticity mechanisms enabled and then blocked inhibitory plasticity during associative recall, we did not find any notable difference in the overall network dynamics compared with the network in which inhibitory plasticity was active (data not shown). These results suggest that inhibitory plasticity is helpful to set inhibition to appropriate levels, but is not necessary to readjust inhibitory weights during recall and delay activity.

## Discussion

We have demonstrated that Hebbian plasticity becomes intrinsically stable by including two or more local forms of non-Hebbian plasticity. In a spiking recurrent neural network model, the orchestrated interplay of these plasticity rules with STP enables the stable formation and recall of cell assemblies. The removal of any one of these mechanisms impaired network function as a memory module. Our results indicate that multiple plasticity mechanisms encountered in the brain work in symphony to enable memory function.

In our model, excitatory synapses are subject to multiple plasticity rules that act on different timescales. First, on the timescale of hundreds of milliseconds, the nonlinear influence of STP on the effective input–output relation of cell assemblies contributes to firing rate bistability at low and intermediate rates. To achieve firing rate bistability through inhibitory feedback^11^ requires fine tuning and spike frequency adaptation (SFA) alone can also be excluded as an alternative because of its overall linearizing effect on neuronal f–I curves.

Second, on the timescale of seconds to minutes, triplet STDP^30^ acts as a plausible form of homosynaptic Hebbian STDP. It is complemented by two non-Hebbian forms of plasticity that act on the same timescale. First, at low firing rates, transmitter-induced potentiation compensates the effects of homosynaptic LTD and ensures a baseline level of activity in the network. Second, at high rates, heterosynaptic depression of strong synapses counteracts LTP and prevents synaptic growth.

Heterosynaptic plasticity in the model is triggered by postsynaptic burst firing, which is in qualitative agreement with experimental findings^21^,^58^. The interaction of heterosynaptic plasticity with consolidation implies that each synapse in our model ‘remembers' its baseline value so that induced synaptic changes can be reverted during a time window of up to 20–60 min (ref. 58). This is achieved by a bistable time-dependent reference weight 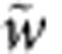 that follows the synaptic weight *w* on a much longer timescale than that of induction of plasticity^59^. In our model, depending on the relative difference of the synaptic weight *w* and the reference weight 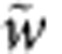, heterosynaptic plasticity can be induced bi-directionally, which provides a possible explanation of experimental data^21^,^49^,^58^.

From the plethora of plasticity phenomena in biological networks we incorporated a small subset into our model. It is likely that some plasticity mechanisms could be replaced by others or that their role could be fulfilled by multiple redundant parallel plasticity pathways. For example, the role of transmitter-induced plasticity could be taken over by fast forms of synaptic scaling^60^. Regardless of the exact identity of the underlying mechanism, we expect two important requirements to be met in any case. First, unstable (Hebbian) forms of plasticity are complemented with compensatory (non-Hebbian) forms of plasticity acting on the same timescale^21^,^42^. Second, to create multiple stable fixed points in long-term plasticity dynamics, compensatory plasticity mechanisms must exist that set in at high postsynaptic activity (*cf.* equation (1)). Experimentally, such mechanisms would reveal their presence as burst detector with a resetting effect^58^ (for example, depotentiation). In our model, this is implemented as a high-power term in the postsynaptic activity (*cf*. equation (1)), but models with a binary activity threshold also fall into this category^49^.

Our model relies on fast forms of plasticity that are able to compensate each other. Slow homeostatic processes were not necessary, when the initial parameters were set to sensible values through manual parameter tuning. Homeostasis ensured network functionality over a broader range of parameter values, exemplified here by homeostatic metaplasticity^30^,^40^ (*cf.* Supplementary Figs 6 and 7), which pushed the network dynamics to an activity regime were cell assemblies emerged naturally. Owing to the intrinsic stability of plasticity, homeostasis in our model acts on the slow timescale observed in experiments^29^,^56^. On a similarly slow timescale, synapses in our model change their internal state, which we called the ‘reference weight'. In our model, this consolidation mechanism is crucial for stabilizing associations between cells. We expect this, or similar, consolidation mechanisms to play an important role for memory capacity^61^.

Inhibitory synapses in our model were subject to a hypothetical form of inhibitory STDP, which is modulated by a global secreted factor^35^. During high global activity this plasticity rule is Hebbian and, similarly to previous work^62^, stabilizes network activity. If, however, global activity is too low, global modulation changes the rule to anti-Hebbian such that it becomes reminiscent of ref. 50. In contrast to ref. 62 the proposed learning rule does not enforce a target firing rate for individual neurons, but rather a network-wide constraint, which is consistent with the notion that inhibitory homeostatic plasticity is modulated by global factors^35^ rather than individual neuronal activity. The fact that, for sensible parameter choices, delay activity in our model emerges also without inhibitory plasticity (*cf.* Supplementary Fig. 5d) suggests that inhibitory plasticity plays a homeostatic role.

Despite its ability to capture the stable formation and recall of cell assemblies, our model has several shortcomings. First, our plasticity model does not capture depotentiation induced by low-activity LTD-like protocols^63^; however, extensions of the model in this direction are possible. Second, our network model does not exhibit a global background state; however, one of the memories is always active. While in some simulations we observed the emergence of a background-like attractor state that did not correlate with any of the learned patterns, the systematic investigation of mechanisms to generate a stable background state is beyond the scope of the present study.

The emergence of cell assemblies through Hebbian synaptic plasticity has been studied in the past^14^,^41^,^57^. Yet, stable learning and recall of memories without run-away of firing rates or the erasure of stored information has been challenging. While ref. 41 illustrated that cell assemblies can be learned in recurrent network models using a plausible learning rule, the model did not produce sufficient self-feedback to actively recall patterns. Mongillo *et al*.^14^ first illustrated assembly formation and recall in larger networks and highlighted the importance of short-term depression to prevent implausibly high firing rates. The work employed a phenomenological model of Hebbian plasticity, in which weights are binary and switch on the timescale of tens of milliseconds. Finally, Litwin-Kumar *et al*.^57^ studied assembly formation and recall in large networks with plausible models of plasticity induction, but focused on transient recall and spontaneous switching between memory items on short timescales (hundreds of milliseconds). For stability this model required rapid homeostatic synaptic scaling on the timescale of seconds.

Several aspects of our model can be tested in experiments. First, in the current form, our plasticity rule links depotentiation with heterosynaptic plasticity. This aspect could be tested in induction experiments of LTP or LTD, which are shortly followed (<10 min) by a ‘post only' burst protocol. Our model predicts an at least partial reversal of plasticity in the induction pathway, but no or only very little change at other synapses. Second, the reversal effect should disappear for intervals between induction and reversal longer than the timescale of consolidation. Third, from the interaction between heterosynaptic plasticity with Hebbian plasticity in our model, we predict the existence of a presynaptic activity threshold for the induction of LTP in protocols with high postsynaptic activity. That is, LTP will only occur when high postsynaptic activity is paired with high presynaptic activity. This behaviour should be robust even if the duration of the protocol is chosen to saturate the synaptic change. In contrast to that, current models of plasticity predict a mere modulation of rate of change with presynaptic activity. Finally, our model states that inhibitory plasticity should be modulated globally by secreted factors to normalize activity at the network level.

In summary, we have shown that the combination of multiple forms of plasticity leads, in an unsupervised manner, to the emergence of stable associative memories in models of spiking recurrent neural networks. Recall of stored memories was demonstrated by selective delay activity. Different forms of plasticity stabilize each other by interacting on comparable timescales in a push–pull manner. Slow homeostatic processes facilitate the learning process, by driving the network and plasticity rules to a suitable working point. Overall, our results highlight the importance of non-Hebbian, rapidly induced forms of plasticity that complement and stabilize Hebbian plasticity of excitatory synapses. Inhibitory as well as globally modulated forms of plasticity will deserve further study, both experimentally and computationally.

## Methods

To study the formation and recall of cell assemblies, we simulated spiking neural network models with sparse random connectivity and multiple forms of synaptic plasticity. The networks we studied consisted of 5,120 integrate-and-fire neurons (4,096 excitatory and 1,024 inhibitory). In the following we describe the different elements of the model. For clarity we only quote the most relevant parameters in the text. A complete tabular description of the model is supplied in the Supplementary Information (Supplementary Tables 1 and 2).

### Neuron model

We use leaky integrate-and-fire neurons with SFA, which receive conductance-based synaptic input. The temporal evolution of the membrane voltage *U*_i_ of neuron i is described by


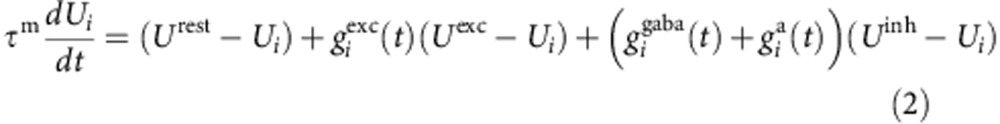


where inhibitory synaptic input 
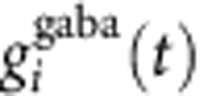
 and a contribution to spike-triggered adaptation 
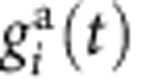
 evolve according to


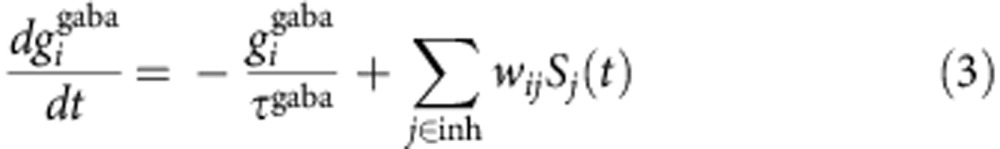



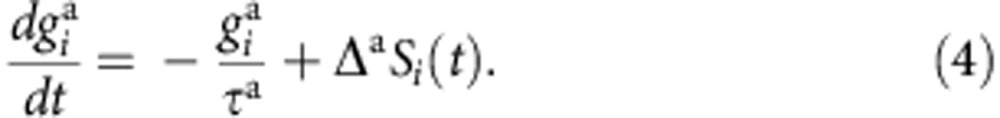


The value of 
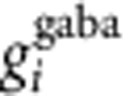
 jumps by an amount *w*_*ij*_ at the moment of spike arrival from presynaptic inhibitory neurons 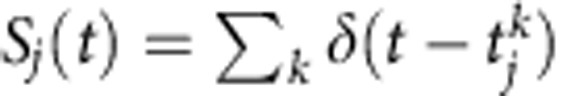 where *δ* denotes the Dirac *δ*-function and 
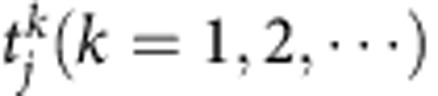
 are firing times of neuron *j*. Analogously, 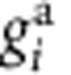 jumps at the occurrence of postsynaptic action potentials *S*_*i*_(*t*) by Δ^a^, the strength of adaptation.

Where this is mentioned explicitly (see Fig. 4c) we add a second adaptation variable with the same temporal evolution as in equation (4), but with different values for Δ^a^ and 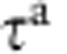. The resulting long-lasting adaptation^54^ mimics cellular behaviour.

Depolarizing current in equation (2) results from excitatory synaptic input


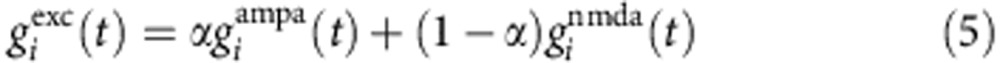


with a fast AMPA-like component 
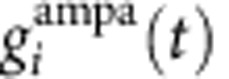
 and a slowly rising and decaying NMDA-like component 
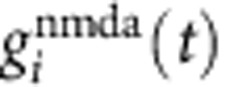
. Their temporal evolution is given by


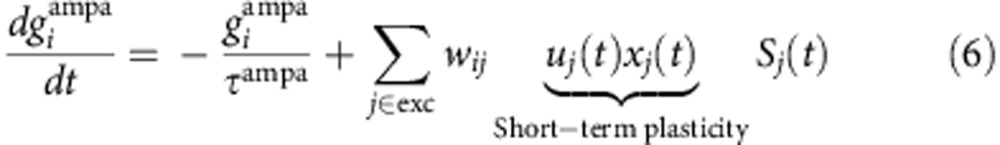



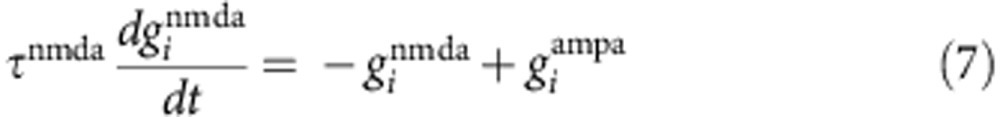


where 
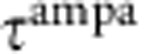
 and 
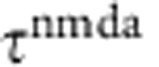
 are time constants and the variables *u*_*j*_(*t*) and *x*_*j*_(*t*) describe the state of STP (see below).

An action potential is triggered when the membrane voltage of neuron *i* rises above the threshold value 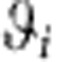. Following a spike the voltage *U*_*i*_ is reset to 
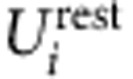
. At the same time, the threshold 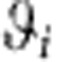 is transiently increased 
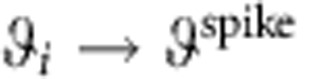
 to implement refractoriness. In the absence of further spikes the dynamic threshold 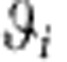 relaxes to its resting state 
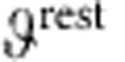



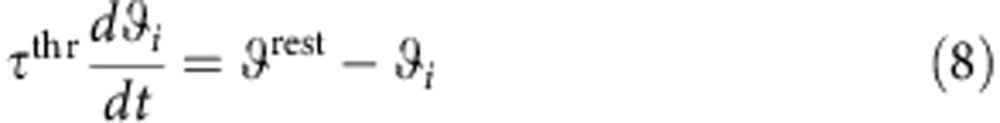


with time constant 
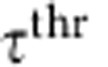
.

### Synaptic plasticity

Our model combines different forms of plasticity. Excitatory synapses exhibit STP, STDP, heterosynaptic plasticity and transmitter-induced plasticity. Inhibitory synapses on excitatory neurons are plastic and obey an STDP rule that is globally modulated by a secreted factor.

### Short-term plasticity

All excitatory connections in our model exhibit STP^10^,^28^. The temporal evolution of the STP state variables *u*_*i*_(*t*) and *x*_*i*_(*t*) (*cf.* equation (6)) is described by


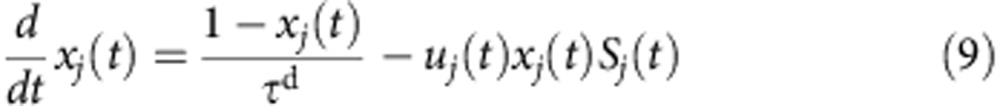



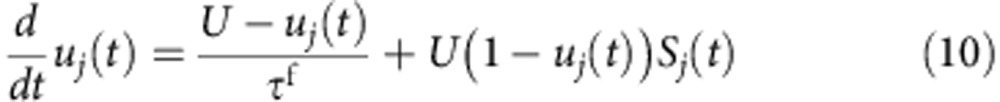


with a fixed parameter set for all synapses (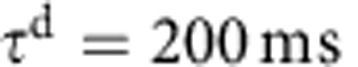, 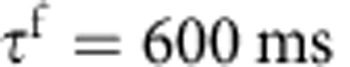 and *U*=0.2).

### Long-term plasticity of excitatory synapses

Plastic excitatory connections are subject to three different plasticity mechanisms: triplet STDP^30^ as well as transmitter-induced^55^ and heterosynaptic plasticity^21^. All three forms of plasticity affect the synaptic weights *w*_*ij*_ directly as follows


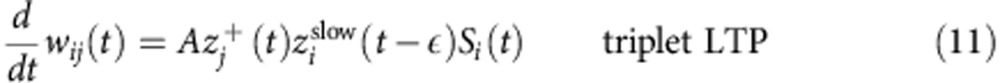



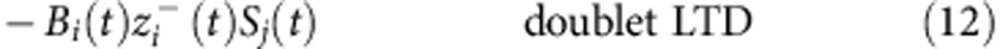



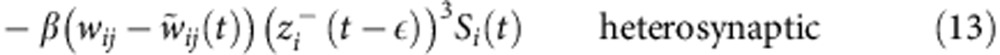



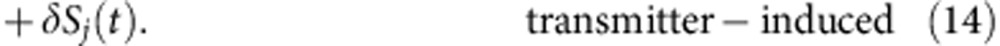


The first two expressions (Expressions (11) and (12)) correspond to the published triplet STDP model^30^. The high power of the postsynaptic firing rate in Expression (13) acts as a burst detector that implements heterosynaptic plasticity. Finally, Expression (14) represents the term responsible for transmitter-induced plasticity. The parameters *A*, *β* and *δ* are fixed. Moreover, *B*_*i*_(*t*)=*A* (unless mentioned otherwise) and the reference weights 
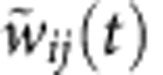
 evolve according to their own dynamics (see below). All occurrences of the state variable 
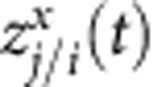
 denote synaptic traces that can occur as pre- or postsynaptic quantities. The offset in the time argument ensures that the current action potential is not counted in the trace. Each synaptic trace evolves independently according to the following differential equation


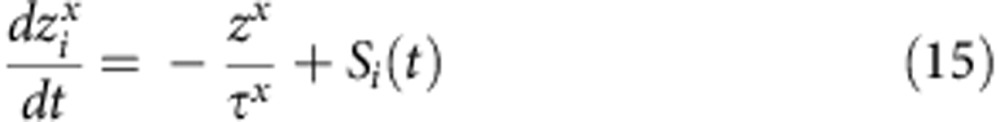


with individual time constants 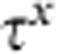. In all simulations the synaptic weight *w*_*ij*_ was additionally algorithmically constrained to the interval 0≤*w*_*ij*_≤5. However, unless heterosynaptic plasticity was turned off (*β*=0; *cf.* Supplementary Fig. 5c), synaptic weights never reached the upper bound.

### Consolidation dynamics

Similar to existing work the reference weight 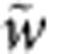 follows the negative gradient of a double well potential^23^,^48^,^59^,^64^. Its evolution is driven by the difference between current weight *w*_*ij*_ and 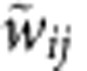 as described by the following expression


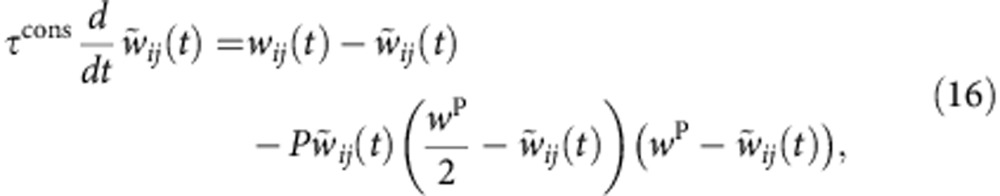


in which the parameter *P* controls the strength of the double-well potential. For 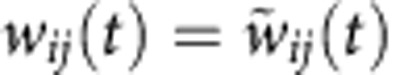, *w*^P^=0.5 defines the upper stable fixed point and the lower stable fixed point lies at 
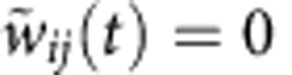
. If *w*_*ij*_ is slightly larger than 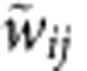, the values of the two stable fixed points of 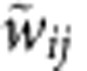 increase. For 
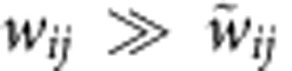
 only one fixed point at a high value remains. Finally, 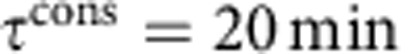 characterizes the rate of convergence towards a stable equilibrium point.

### Homeostatic regulation of LTD

In most simulations, we keep the rate *B*_*i*_(*t*) of LTD fixed (*B*_*i*_(*t*)=*A*) and choose initial synaptic strength such that a subset of neurons responds with rates above the LTP threshold to external stimulation. We thereby implicitly assume that the network has been prepared in this state by one or multiple homeostatic mechanisms that act on a much longer timescale than those captured in our simulations. To test whether homeostasis could indeed achieve such parameter tuning, we ran a subset of simulations in which *B*_*i*_(*t*) was explicitly time-dependent^30^,^40^ (Supplementary Figs 6 and 7). Where homeostatic regulation of LTD is mentioned explicitly in the text, we set


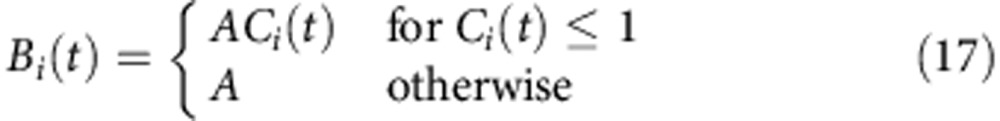



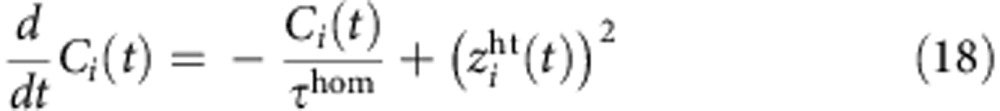


with 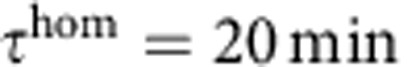, in which 
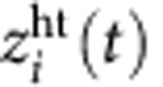
 is another synaptic trace as in equation (15) with 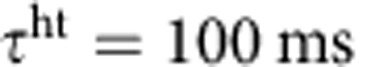. The ‘if' condition in equation (17) ensures that LTD cannot remove the rate fixed point associated with the elevated activity in a cell assembly. The homeostatic control of LTD as implemented by equations (17) and (18) ensures that in a situation where all excitatory cells in a network remain for a long time at a rate of 1 Hz or below, the plasticity threshold (*cf*. equation (1)) is reduced until some cells start to respond at elevated rates.

### Long-term plasticity of inhibitory synapses

While it is widely accepted that synaptic inhibition in neural networks controls the overall network activity, it is less clear how inhibitory connections are formed and how the strength of inhibition adapts. In our model we use a hypothetical form of inhibitory synaptic plasticity, which is modulated by a global secreted factor^35^. Roughly speaking, inhibitory synapses tend to be potentiated whenever the global network activity is too high.

Specifically, inhibitory synapses on excitatory neurons in our model obey the following STDP rule


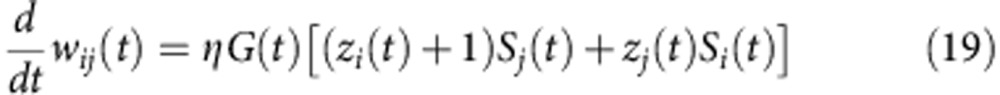


where *η* is a constant, the *z*_*j/i*_ denote pre/postsynaptic traces with common time constant 
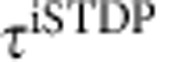
 (*cf.* equation (15)), *S*_*j/i*_(*t*) are the pre/postsynaptic spike trains and *G*(*t*) is a quantity that linearly depends on the global secreted factor *H*(*t*). In particular, we set 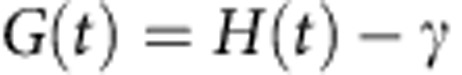, where *H*(*t*) is defined as the low-pass-filtered version of all spikes in the excitatory population given by


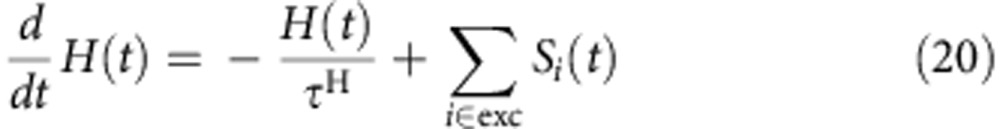


with characteristic time constant 
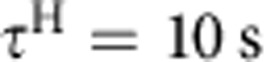
. We interpret *H*(*t*) as a chemical signal that neurons secrete when they are active and that diffuses in the extracellular space where it can be sensed by other neurons or synapses as a measure of the global network activity. When this activity drops below its target value 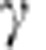, *G*(*t*) is smaller than zero and the STDP learning rule (equation (19)) becomes a unidirectional ‘depression only' learning rule^50^,^65^. Conversely, if the network activity is higher than 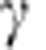 the learning rule becomes Hebbian. Similar to previous theoretical work^62^ this has a stabilizing effect on the overall network dynamics.

### Simulation of postsynaptic tetanization protocols

To simulate the postsynaptic tetanization protocol^49^ (*cf.* Fig. 5a), we connected a single postsynaptic neuron with 1,000 presynaptic connections endowed with heterosynaptic and transmitter-induced plasticity as well as triplet STDP. We used two independent random initial values for the synaptic weights *w*_*ij*_ and their reference values 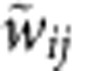 (equations (11)–(16), 36, 37, 38, 41, 45). Both were drawn independently from a normal distribution with mean 0.3 and s.d. 0.3. To ensure positive values after the assignment, all weight values below zero were then set to zero.

We simulated the ongoing measurement of the EPSP size from two different pathways (Fig. 5ai,ii), which were stimulated alternatingly with one spike each (50-ms time difference) every 7.5 s. This stimulation was maintained during the entire protocol except during tetanization (10 min<*t*<13 min) where the postsynaptic cell was forced to spike through simulated current injection (three trains with 1-min offset consisting of 10 burst at 1 Hz with five spikes at 100 Hz each^49^).

### Stimulation paradigm

For simulations requiring external stimulation we used the following paradigm. In the absence of a stimulus all input neurons were firing with Poisson statistics at a fixed rate of 10 Hz. A designated set of stimuli was fixed at the beginning of the simulation as a graded (Fig. 2e) or a binary activation pattern of input neurons (for example, Fig. 3a). During stimulation, input cells that were active in a given pattern fired with a by *ζ* × 35 Hz increased rate (unless mentioned otherwise), in which *ζ* was one for binary patterns or from the interval [0,1] for graded activity patterns.

Stimulus order was chosen randomly with equal probability for all stimuli unless mentioned otherwise. The interstimulus intervals and stimulus durations were drawn from exponential distributions with the mean values *T*_Off_ and *T*_On_, respectively. During an initial burn-in period of at least 50 s no stimulation was given. For all network simulations we initially set *T*_On_=1 s, while *T*_Off_ was set to *T*_Off_=2 s. To test for delay activity, these values were switched to *T*_On_=0.2 s and *T*_Off_=20 s after 1 h of simulated time. In all cases, the same network simulation was simulated continuously to first learn a set of stimuli (*t*<1 h) to test for delay activity (1 h<*t*<2 h) and for associativity (2 h<*t*). All forms of plasticity were permanently active during the entire simulation, unless blockage is explicitly mentioned.

### Details of feed-forward network simulations

To characterize the effect of our excitatory plasticity rule on a single postsynaptic neuron, we simulated two simple feed-forward networks without inhibition (Fig. 2b,c; Supplementary Fig. 3). More precisely, we simulated a single postsynaptic neuron that received 80 plastic excitatory input from each of two populations of Poisson neurons. The neurons in one population fired at 10 Hz and the initial weight *w*_0_ took different values in the interval (0.2,0.35) as stated in Fig. 2b. Neurons in the second Poisson population fired at 1 Hz and the initial weight was initialized at a value of *w*_ctl_(*t*=0)=0.1.

In Fig. 2e we used a similar set-up with 1,000 presynaptic Poisson inputs (initial weight *w*_0_=0.05 and 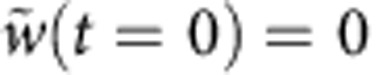) all firing at a constant background rate of 10 Hz. The stimulus set consisted of 10 Gaussian firing rate profiles in the presynaptic index with fixed s.d. *σ*=50, but different centres. Stimulation onset was at *t*=100 s mean stimulation interval *T*_On_=20 s (*T*_Off_=100 ms).

### Balanced network model

We used a balanced network model consisting of 4,096 excitatory and 1,024 inhibitory integrate and fire neurons. The connectivity within the network was random sparse with an overall connection probability of 10%. Neurons in the excitatory population received additional input from an external population of equal size that provided noisy background input. For Figs 3 and 4 these input connections were pre-structured so that cells from within a circular area (radius *R*=8) in the 64 × 64 input space projected to individual network neurons (Fig. 3a). The centre of the circle was chosen randomly within the input space for each postsynaptic neuron. For Supplementary Figs 6 and 7 input connections were initialized with random sparse connectivity with 5% connection probability. All excitatory afferent connections relayed stimuli from the external Poisson input population to the network (see ‘Stimulation paradigm' above).

In simulations involving plasticity, all afferent connections to excitatory cells in the network were plastic. Moreover, plasticity was always active, also during periods when the network was cued with partial stimuli (for example, Fig. 4).

### Directed stimulation of two neural subpopulations

To directly stimulate two subsets of neurons (Fig. 6) we proceeded as follows. To assign the subsets, all cells from the reserve pool (*cf.* black bar in Fig. 3h) plus 200 neurons coding for other patterns were split into two approximately equal populations (R1/R2). At *t*=2 h (*cf.* Fig. 6a) input from existing input synapses was turned off and synaptic input from a different input modality was simulated by driving neurons in each population (R1/R2) through static synapses (*w*=0.2, no STP) from two independent external populations of Poisson neurons (firing at 10 Hz) of equal size as R1 and R2, respectively. Individual neurons within R1 and R2 received connections from the corresponding external pools with a fixed connection probability of 5%. To stimulate R1 and R2, we used the same stimulation paradigm as before with *T*_On_=1 s and *T*_Off_=5 s during the first hour (2 h<*t*<3 h) and *T*_On_=0.2 s and *T*_Off_=20 s during the second hour (3 h<*t*<4 h) to illustrate the stability of working memory states. At *t*=4 h the input protocol was reverted to the original input synapses.

### Continuous recall of only one assembly over hours

To make one assembly constantly active (Fig. 7), we switched off stimulation with external cues. To avoid any spontaneous activation of other assemblies during multiple hours of simulated time, we reduced the firing rate of neurons in inactive assemblies to ∼0.5 Hz by reducing the value of the parameter *δ* of transmitter-induced plasticity (*δ*=1 × 10^−5^). This manipulation did not have a notable effect on recall dynamics (Fig. 3a,b; first two hours), but completely abandoned spontaneous state transitions within the time frame of our simulation.

### Simulation details and code

All simulation codes were written in C++ and are based on the network simulation framework Auryn^66^. The code is freely available on the author's GitHub page^67^. Neuronal state variables were updated using the forward Euler method with 0.1-ms temporal resolution. The only exception from that was the slow evolution of the reference weights 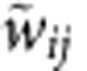, which were updated with a time step of 1.2 s for efficiency reasons.

### Determining readout populations and overlap

To determine which cells respond to a given stimulus *μ*, we compute the stimulus-evoked firing rate 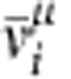 of neuron *i* from spiking data in the interval 3,000 s<*t*<3,500 s. We count neuron *i* as coding for stimulus *μ* if 
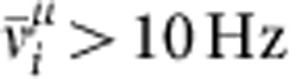
. To characterize differences in evoked network responses to different stimuli, we plot the covariance matrix (for example, Fig. 3f,g)


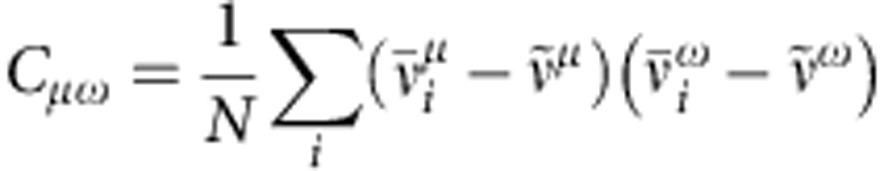


where 
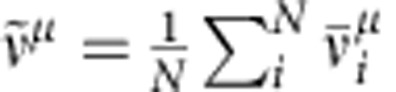
 is the mean evoked population response of all neurons for stimulus *μ*.

From the set *M*_*μ*_ of neurons responding to stimulus *μ* (that is, all neurons *i* with 
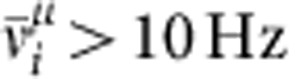
) we compute the ‘activity', that is, the population firing rate in the set as


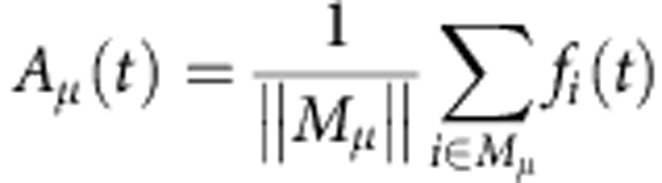


where 
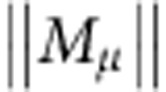
 is the number of neurons in the set *M*_*μ*_ and *f*_*i*_ is the firing rate of neuron *i* with a temporal resolution of 50 ms. These data are either plotted directly as activity along with spike raster plots (for example, Fig. 3d,j) or used to compute peristimulus time histograms (for example, Fig. 4b).

## Additional information

**How to cite this article:** Zenke, F. *et al*. Diverse synaptic plasticity mechanisms orchestrated to form and retrieve memories in spiking neural networks. *Nat. Commun.* 6:6922 doi: 10.1038/ncomms7922 (2015).

## Supplementary Material

Supplementary Information Supplementary Figures 1-9, Supplementary Tables 1-2, Supplementary Methods and Supplementary References

Supplementary Movie 1 The video demonstrates on-line learning in a recurrent spiking network model through orchestrated learning rules (cf. Figure 3). Plasticity is active at all times.
