# Supplementary material for: Diverse synaptic plasticity mechanisms orchestrated to form and retrieve memories in spiking neural networks
Source: Nat Commun. 2015 Apr 21;6:6922. doi: 10.1038/ncomms7922 (PMC4411307; doi:10.1038/ncomms7922)
Supplement: Supplementary Information — Supplementary Figures 1-9, Supplementary Tables 1-2, Supplementary Methods and Supplementary References [file ncomms7922-s1.pdf]

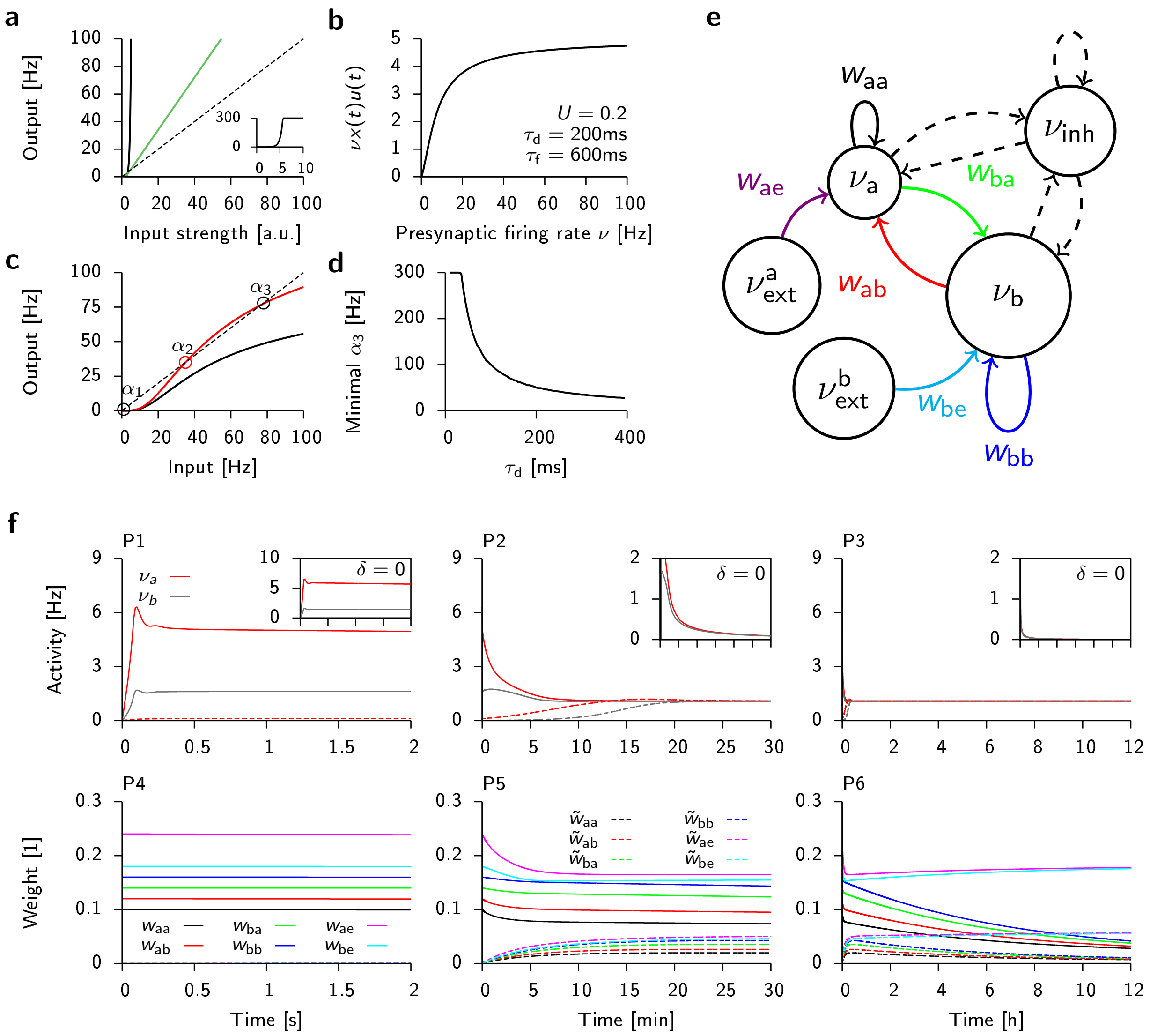
a
Output [Hz]
Input strength [a.u.]
300
0
0 5 10
b
νx(t)u(t)
Presynaptic firing rate ν [Hz]
U = 0.2
τd = 200ms
τf = 600ms
c
Output [Hz]
Input [Hz]
α1
α2
α3
d
Minimal α3 [Hz]
τd [ms]
e
waa
νinh
νa
wae
wba
ν^a_ext
wab
νb
ν^b_ext
wbe
wbb
f
P1
P2
P3
Activity [Hz]
νa
νb
δ = 0
P4
P5
P6
Weight [1]
waa
wab
wba
wbb
wae
wbe
w̃aa
w̃ab
w̃ba
w̃bb
w̃ae
w̃be
Time [s]
Time [min]
Time [h]

Supplementary Figure 1 – Stability of orchestrated learning rules is captured by a simple rate model. (**a**) Schematic representation of the typical input output relation of a neuron without spike frequency adaptation (SFA) shown in black and with SFA (green). Above threshold, the rise is linear (green) or super-linear (black). Diagonal given in dashed black for reference. Saturation occurs at the maximum firing rate of 300 Hz (inset). (**b**) Steady state synaptic transfer function for short-term plasticity[1] (STP) with Poisson input. Synaptic depression leads to a pronounced saturation of synaptic transmission at ≈30 Hz. (**c**) Combined input-output relation g of a non-adapting rate neurons from **a** with STP transfer function from **c** (black) and excitatory self-feedback $w_{\mathrm{ee}} = 1.9$ (black) and $w_{\mathrm{ee}} = 2.0$ (red). The sigmoidal shape of the effective transfer function gives rise to three intersection points $(\alpha_1, \alpha_2, \alpha_3)$ with the diagonal (dashed) in the regime < 100 Hz. (**d**) Lowest possible firing rate at fixed point α_3 as a function of the time constant τ_{d} of recovery from synaptic depression. If synaptic depression vanishes ($\tau_{\mathrm{d}} \to 0$), the firing rate inside an active assembly is close to 300 Hz[1]. (**e**) Schematic representation of the rate model. The excitatory network neurons are split into two populations. ν_{a} describes the firing rate of the "assembly" neurons, whereas ν_{b} characterizes the rate of the "background" neurons. Both populations receive external input from external populations $\nu_{\mathrm{ext}}^{\mathrm{a}}$ and $\nu_{\mathrm{ext}}^{\mathrm{b}}$ respectively. Finally, the excitatory populations project to and receive inhibitory input from a shared pool of inhibitory neurons characterized by the firing rate ν_{inh}. All external and excitatory-to-excitatory synaptic connections are modeled as plastic with orchestrated plasticity rules. For simplicity, connections from and to the inhibitory population are taken as static. (**f**) Evolution of firing rate (top row) and weight dynamics (bottom row) of the rate model on three different timescales (columns) for constant input $\nu_{\mathrm{ext}}^{\mathrm{a}} = \nu_{\mathrm{ext}}^{\mathrm{b}} = 10$ Hz. The dashed lines in the top row show the rate dynamics for the same network but with different initial conditions. The insets in the top row show the same network, but without transmitter induced plasticity ($\delta = 0$), which leads to a decay of firing rates to zero. The bottom row shows weight dynamics for one initial state of the network. Solid lines represent the synaptic weights as colored in **a**. The dashed lines represent the evolution of the respective reference weights $\tilde{w}$ which were all initialized at zero in this simulation. w_{ae} and w_{be} converge to higher values than w_{aa}, w_{bb}, w_{ab}, or w_{ba}, because the external input pools fire at higher rates (10 Hz) than the stationary rates ν_{a} and ν_{b} of the two excitatory populations. For $\delta = 0$ the firing rates converge to zero.

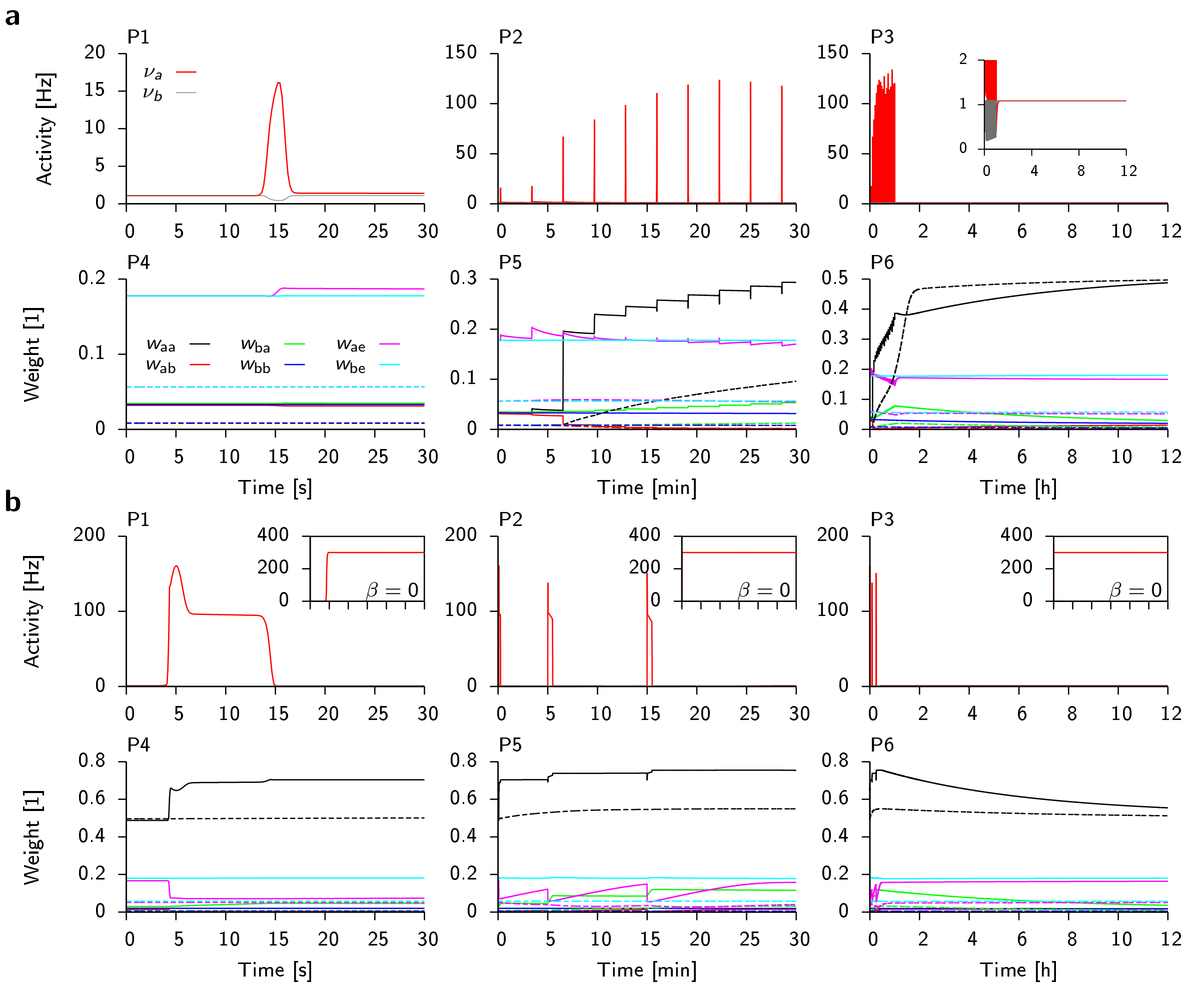

Supplementary Figure 2 – Learning and recall dynamics in a rate model with orchestrated learning rules. (**a**) Same as Supplementary Fig. 1 f, but for a protocol in which population ν_a receives repeated external high frequency stimulation (20 Gaussian rate pulses equally spaced over the interval [15 s,1 h]). The inset in panel P3 illustrates that firing activity is non-zero (≈ 1 Hz) in the absence of stimulation. The weight w_{aa} (solid black) within the assembly and its corresponding reference weight $\tilde{w}_{aa}$ (dashed black) converge to stable hight values of approximately 0.5. (**b**) Same as above, but during recall dynamics. The assembly population receives brief excitatory stimuli (Gaussian rate profile; $\sigma = 0.5$ s) at times 5 s, 300 s and 900 s which are each followed by an equally brief inhibitory stimulus (times: 15 s, 330 s and 930 s). The insets in the top row for a network without heterosynaptic plasticity ($\beta = 0$), show that firing rates immediately explode and saturate at the maximum firing rate of 300 Hz, whereas in the normal network retrieval happens at rates of about 100 Hz and is stopped by the inhibitory pulses.

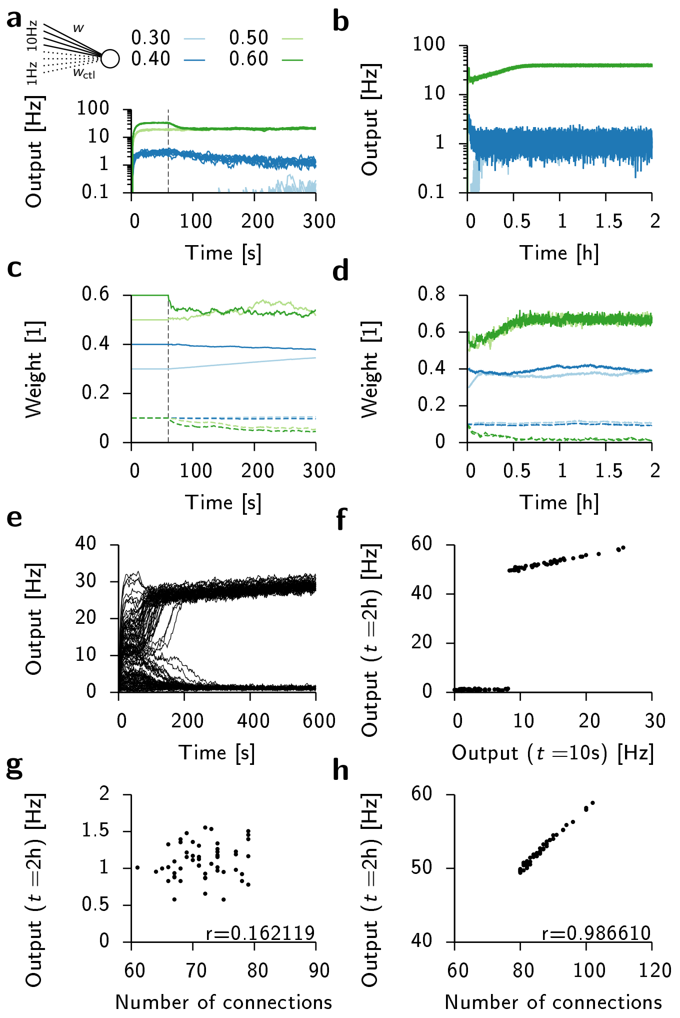

Supplementary Figure 3 – Firing rates are bistable under triplet STDP with transmitter-induced and heterosynaptic plasticity. (**a**) Mean firing rates during 300 s for 100 simulations as shown in Figure 2 b. Different colors signify four different initial conditions of the afferent synaptic weights. Long-term plasticity is enabled in the model after an initial period of 60s (dashed line). (**b**) Same as **a** but on the timescale of 2h. (**c**) Typical evolution of individual synaptic weights in the simulation shown in **a**. The different initial conditions are indicated in color as in **a**. Solid lines code for a weight in the 10Hz pathway, dashed lines for a weight in the 1Hz control pathway. The asymptotic value in the control pathway depends on the firing rate of the postsynaptic neuron via heterosynaptic plasticity. (**d**) Same as **c** but on a longer timescale. (**e**) Same as **a** for 100 simulation runs with fixed initial weight value, but with varying numbers of connections, drawn randomly with fixed connection probability of 10 % between presynaptic Poisson neurons and postsynaptic cell. (**f**) End point firing rates at $t = 2\text{h}$ from **e** vs. initial firing rates ($t = 10\text{s}$). (**g**) End point firing rates as in **f** for the cluster at low rates. r is the correlation coefficient for the data shown. (**h**) End point firing rates as in **f** for the cluster at high rates.

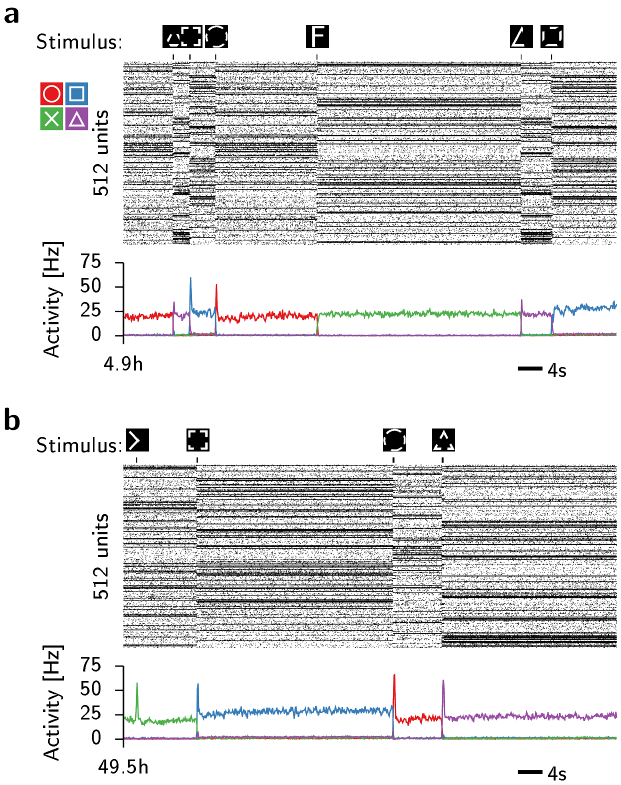

Supplementary Figure 4 – Delay activity is stable over hours to days. (**a**) Network activity during associative recall. Top icons: Partial and distorted cues fed into the network (black bars code for time and duration of the stimulation). Middle panel: Spike raster of network activity. Bottom panel: Population rate of the four acquired assemblies (color code corresponds to the four schematic stimuli shown on the top left). The "F" is a new stimulus the network did not see during training, which causes it to be misclassified as a cross. (**b**) Same as before, but after two days of simulated plastic network activity.

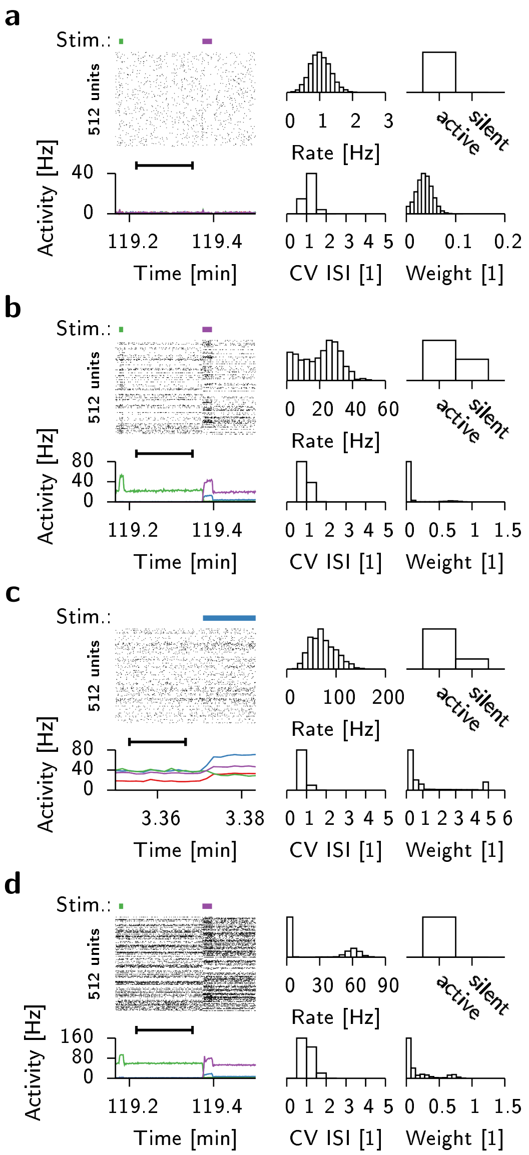

Supplementary Figure 5 – Failure modes arising from blocking single plasticity mechanisms. (**a-d**) Top left: Spike raster (256 excitatory neurons) with stimulation interval and stimulus identity on top (colored bars). Bottom left: Activity in putative memory patters as determined in the simulation shown in Figure 3. Histograms (from top left to bottom right): 1) Neuronal firing rates determined over the interval indicated below the raster plot. For clarity only neurons that fired at least one spike during the interval are considered for the histogram. 2) Bar plot illustrating the relative numbers of active (at least one spike during one hour) and silent neurons. 3) CV ISI distribution computed over the interval indicated below the raster plot on the left. 4) Excitatory synaptic weights as determined at the end of the simulation (at $t = 300\,\mathrm{s}$ for **b**, $t = 2\mathrm{h}$ otherwise). (**a**) Blocked homeostatic metaplasticity of LTD in a network with weak random initial input (cf. Fig. 3). The network fails to develop selectivity. (**b**) Blocking transmitter-induced plasticity causes many neurons to fall and remain silent. (**c**) Blocked heterosynaptic plasticity causes rapidly increasing firing rates without learning or emergence of delay activity (simulation stopped after 300 s). Note the bi-modal weight distribution. A weight value of 5 corresponds to the maximally allowed weight. (**d**) Blocking inhibitory plasticity allows the network to develop delay activity, but at highly elevated firing rates. Compare firing rates (top left histogram) with Fig. 3 k in the main text.

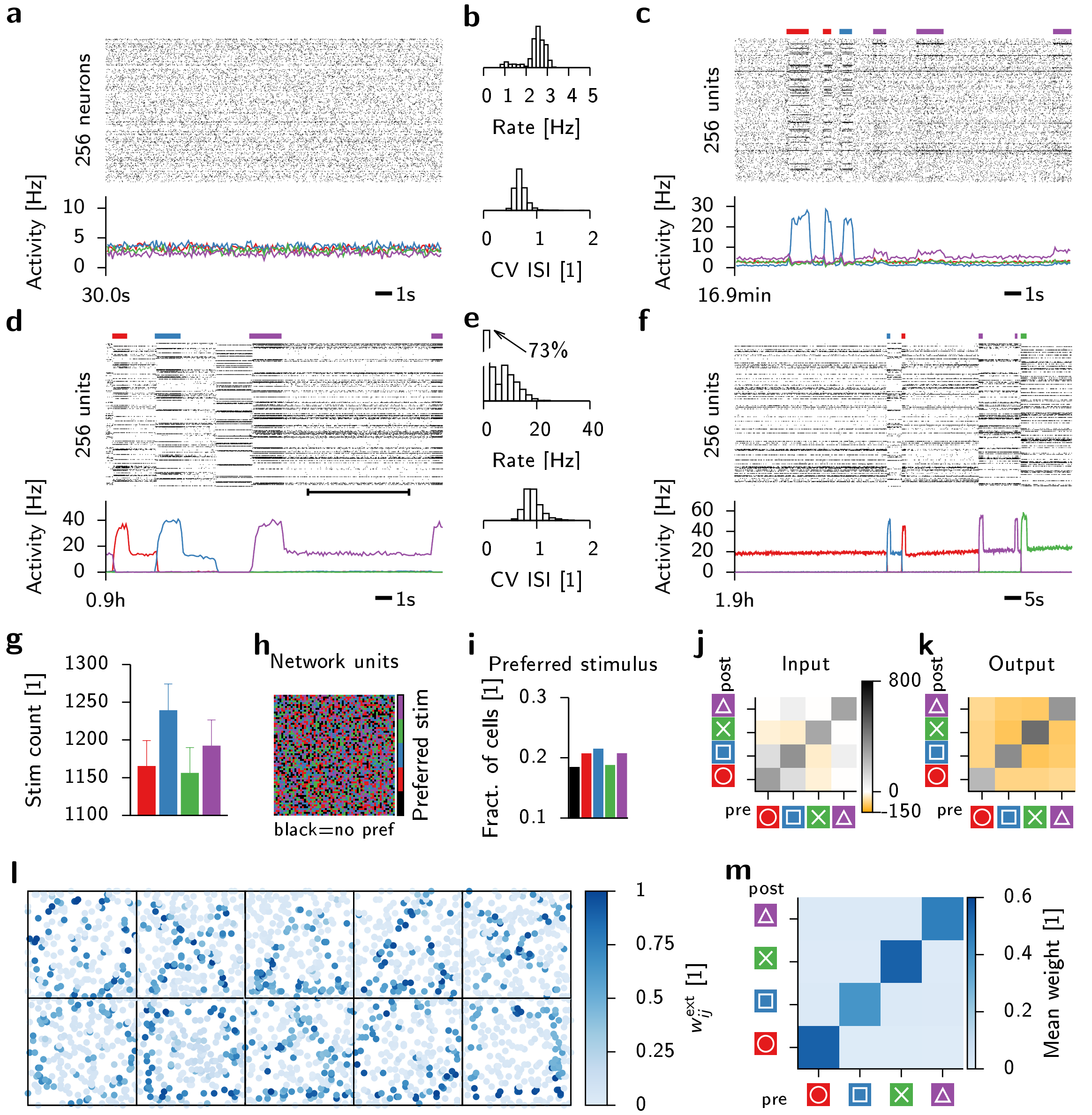

Supplementary Figure 6 – Stable formation and recall of cell assemblies in networks with homeostatic metaplasticity for initially weak and unstructured connectivity. (**a**) Top panel: Spike raster of network activity after 30s of activity. Bottom panel: Population firing rate of cell assemblies corresponding to the four stimuli as determined after one hour of simulation. (**b**) Histograms of network spike statistics during initial activity shown in **a**. Top: Firing rate, bottom: CV ISI. (**c**) As in **a**, but after about 17min of external stimulation. Stimulation times and stimulus identity are indicated by colored bars at the top of the plot. (**d**) Same as **a** after about one hour of simulation. (**e**) Same as **b** but for interval marked in **d**. (**f**) Same as **d** but with increased inter-stimulus-interval ($T_{\mathrm{Off}} = 20\mathrm{s}$). (**g**) Histogram showing the number of stimulus presentations at $t = 1\mathrm{h}$. Error bars indicate square root of the respective value, corresponding to the expected s.d. for rare count events. (**h**) Network neurons laid out on 2D grid with stimulus preference indicated in color (black: no stimulus preference). (**i**) Bar plot displaying the fraction of neurons coding for a specific stimulus (black: no stimulus preference). (**j**) Covariance matrix of the stimulus-evoked firing rates in the Poisson input neurons (Methods). (**k**) Same as **j**, but for observed network activity after learning ($t = 1\mathrm{h}$). For color code see **j**. (**l**) Receptive fields of 10 randomly selected neurons within the network. (**m**) Mean weight strength between neurons ordered according to their preferred stimulus.

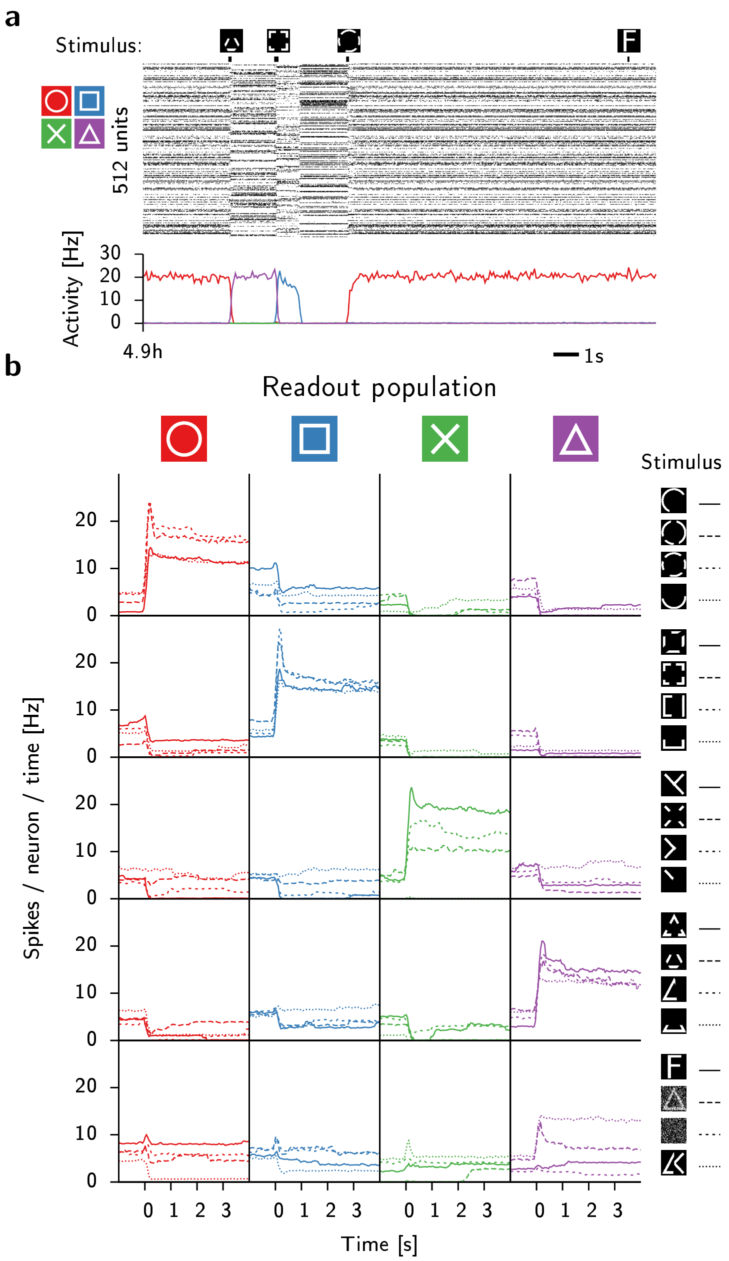

Supplementary Figure 7 – Network states are associative and serve as working memory in the network from Figure 6. (**a**) Top icons: Distorted cues fed into the network (black bars below that code for time and duration of the stimulation). Middle panel: Spike raster of network activity (only every fourth spike of every fourth neuron shown for clarity). Bottom panel: Population rate of the four acquired network states. After presentation of a distorted square the network remains in this state briefly and then spontaneously switches to a network state that does not correspond to any of the previously learned stimuli. (**b**) Population averaged peristimulus time histograms of the four relevant readout populations for all different distorted stimuli presented (keys on the right).

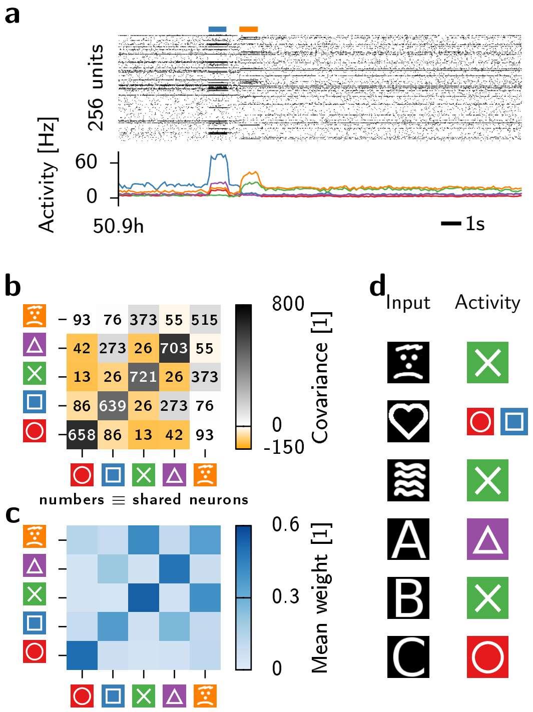

Supplementary Figure 8 – Mere exposure to novel stimuli in a network with previously formed receptive fields does not yield working memory states. (**a**) Spike raster and pattern activity after exposure to a novel stimulus (orange; ≈ 2000 stimulations) through the same set of synapses which had previously learned multiple stimuli (cf. Supplementary Fig. 4). (**b**) Covariance matrix (color coded) of evoked population activity between with previously established stimulus preferences and the novel pattern (grumpy face). The numbers encode the number of units which responded with more than 10 Hz to a single (diagonal) or two patterns (off diagonal). (**c**) Averaged synaptic weights in matrix representation between the four previously learned patterns and the novel pattern. (**d**) Schematic representation of different input patterns (left), presented to the network repeatedly after the initial learning session, and how they were typically classified by the network (right).

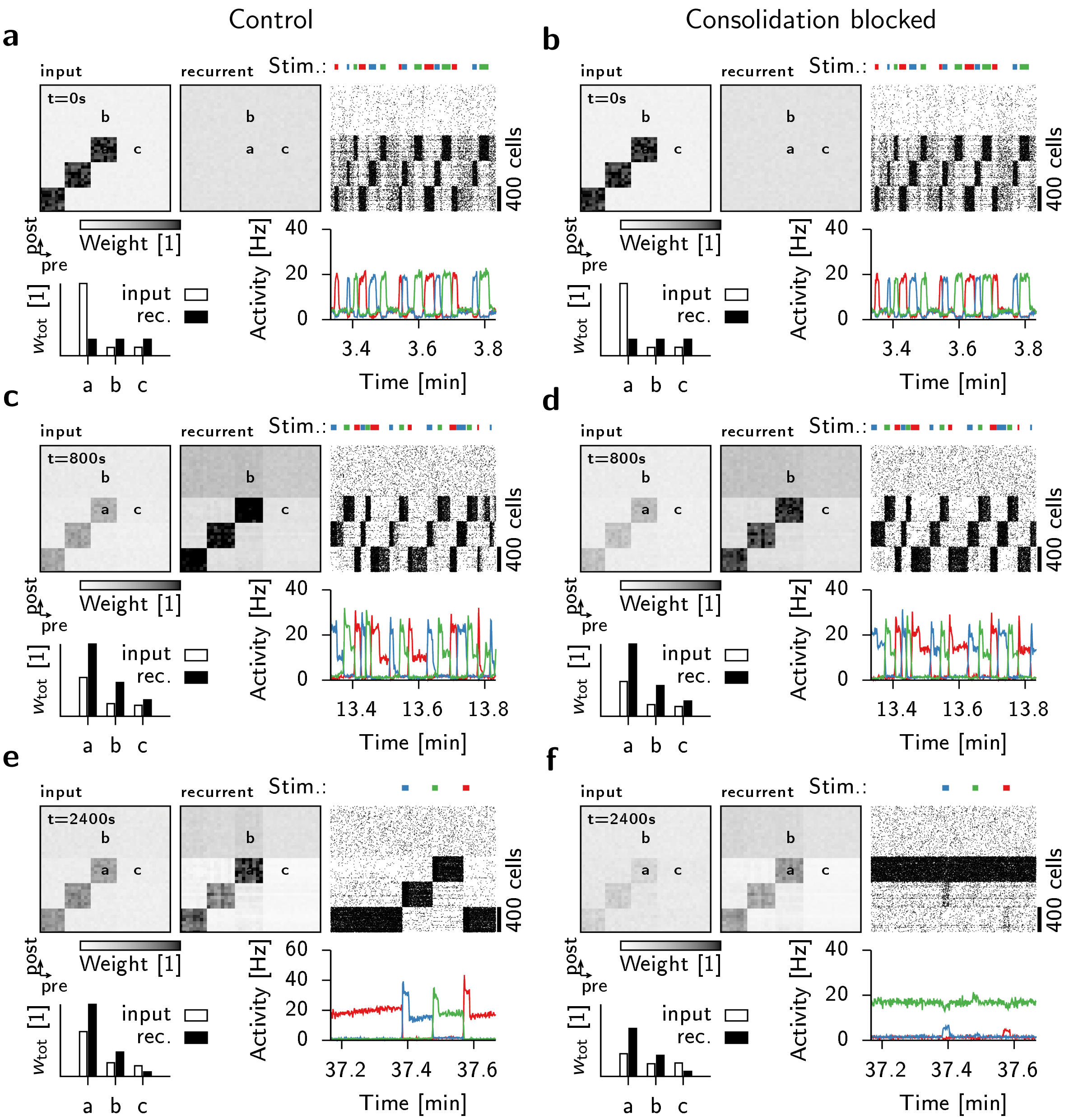

Supplementary Figure 9 – Blocking consolidation causes cell assemblies to decouple from external input. (**a**,**c**,**e**) Control network with consolidation. (**b**,**d**,**f**) Network in which all consolidation dynamics are blocked. (**a**) Top left: Initial state of control network. 2,000×2,000 neuron section of input weight matrix with preset block patterns on diagonal. Second from left: 2,000×2,000 neuron section of recurrent weight matrix without initial structure. Letters a,b,c mark regions used to compute average weight (bar plot, bottom left). Right: Spike raster of the 2,000 network neurons corresponding to the weights shown on the left. The colored bars on the top indicate the stimulus identity. Bottom right: Activity in 3 × 400 neuron block patterns over time. (**b**) Same as **a**, but with consolidation dynamics blocked. Otherwise the network simulation is unchanged. (**c**,**d**) Same as before at later time. After 800 s the recurrent connectivity in both networks has formed three cell assemblies which function as working memory for the last stimulus. (**e**,**f**) After 20min the mean stimulation interval is switched from $T_{\mathrm{Off}} = 2$s to $T_{\mathrm{Off}} = 20$s. (**e**) In the network with consolidation the input connections remain strong enough to trigger reliable state switching. (**f**) As external input weights fade away he network is “stuck” in a single recall state. **e** and **f**: same data as in Figure 8 of main text.

Supplementary Tables

Supplementary Table 1 – Tabular description of network model.

A	Model Summary
**Populations**	Three: excitatory, inhibitory, Poisson input
**Topology**	—
**Connectivity**	Random sparse connectivity (fixed probability)
**Neuron model**	Leaky integrate-and-fire, relative and absolute refractory period, spike-triggered adaptation
**Synapse model**	Conductance based, exponentially decaying (AMPA, GABA), and double exponential (NMDA)
**Plasticity**	Custom models see D2–D4 (below).
**Input**	Poisson input with additional rate coded input patterns
**Measurements**	Spike activity, synaptic efficacy (continuously of a subset of synapses and of all synapses at the end of a simulation)

B		Populations
**Name**	**Size**	**Elements**
E	4,096	Adaptive IF neuron
I	1,024	IF neuron
Stim	4,096	Poisson stimulus population

C			Connectivity
**Name**	**Source**	**Target**	**Pattern**
EE	E	E	Fixed prob. $\epsilon = 0.1$, $w = 0.1$, excitatory, plastic
EI	E	I	Fixed prob. $\epsilon = 0.1$, $w = 0.2$, excitatory
IE	I	E	Fixed prob. $\epsilon = 0.1$, $w = 0.1$, inhibitory, plastic
II	I	I	Fixed prob. $\epsilon = 0.1$, $w = 0.2$, inhibitory
StimE	Stim	E	Fixed prob. $\epsilon = 0.05$, $w = 0.2$, excitatory, plastic
			For all above: Fixed delay $D = 0.8$ms

D1	Neuron and Synapse Model
**Name**	AIF neuron, used for all excitatory neurons
**Type**	Adaptive leaky integrate-and-fire, absolute and relative refractoriness, conductance input, spike triggered adaptation, AMPA and GABA-like conductances exponentially decaying, double exponential NMDA-like conductance
**Subthreshold dynamics**	$\tau^{\mathrm{m}} \frac{dU_i}{dt} = (U^{\mathrm{rest}} - U_i) + g_i^{\mathrm{exc}}(t)(U^{\mathrm{exc}} - U_i) + \left(g_i^{\mathrm{gaba}}(t) + g_i^{\mathrm{a}}(t) + g_i^{\mathrm{b}}(t)\right)(U^{\mathrm{inh}} - U_i)$
**Excitation**	$g_i^{\mathrm{exc}}(t) = \alpha g_i^{\mathrm{ampa}}(t) + (1-\alpha) g_i^{\mathrm{nmda}}(t)$ $\frac{d}{dt} g_i^{\mathrm{ampa}} = -\frac{g_i^{\mathrm{ampa}}}{\tau^{\mathrm{ampa}}} + \sum_{j \in \mathrm{exc}} x_j(t) u_j(t) w_{ij} S_j(t)$ for the definition of short term plasticity variables x_j and u_j see D2 below. $\tau^{\mathrm{nmda}} \frac{d}{dt} g_i^{\mathrm{nmda}} = -g_i^{\mathrm{nmda}} + g_i^{\mathrm{ampa}}$
**Inhibition**	$\frac{d}{dt} g_i^{\mathrm{gaba}} = -\frac{g_i^{\mathrm{gaba}}}{\tau^{\mathrm{gaba}}} + \sum_{j \in \mathrm{gaba}} w_{ij} S_j(t)$
**Adaptation**	$\frac{d}{dt} g_i^{\mathrm{a}}(t) = -\frac{g_i^{\mathrm{a}}}{\tau^{\mathrm{a}}} + \Delta^{\mathrm{a}} S_i(t)$ $g_i^{\mathrm{b}}(t) = 0$ except in simulations with adaptation on multiple timescales: $\frac{d}{dt} g_i^{\mathrm{b}}(t) = -\frac{g_i^{\mathrm{b}}}{\tau^{\mathrm{b}}} + \Delta^{\mathrm{b}} S_i(t)$
**Threshold**	$\tau^{\mathrm{thr}} \frac{d}{dt} \vartheta_i(t) = -\vartheta_i(t) + \vartheta^{\mathrm{rest}}$
**Spiking**	If $U_i > \vartheta_i(t)$ then $S_i(t) \to S_i(t) + \delta(t)$ (emit a spike) and $\vartheta_i(t) \to +50\mathrm{mV}$ and $U_i \to U^{\mathrm{rest}}$

**Name**	IF neuron, used for all inhibitory neurons
**Type**	The non-adaptive inhibitory neurons were implemented identically except of the omission of the adaptation terms $g_i^a = g_i^b = 0$.

D2	Plasticity Model
**Name**	Short term plasticity following[2].
**Type**	Depressing and facilitating short term dynamics
**Acts on**	EE,EI,StimE
**Dynamics**	$\frac{d}{dt} x_j(t) = \frac{1 - x_j(t)}{\tau^{\mathrm{d}}} - u_j(t)\, x_j(t)\, S_j(t)$ $\frac{d}{dt} u_j(t) = \frac{U - u_j(t)}{\tau^{\mathrm{f}}} + U\,(1 - u_j(t))\, S_j(t)$

D3	Plasticity Model
**Name**	Plasticity rule for excitatory synapses
**Type**	Triplet STDP rule with pre and post offset terms and slower consolidation dynamics
**Acts on**	EE and StimE
**Synaptic traces**	$\frac{d}{dt} z_j^+(t) = -\frac{z_j^+(t)}{\tau^+} + S_j(t)$ with presynaptic spike train $S_j(t)$.<br>$\frac{d}{dt} z_i^-(t) = -\frac{z_i^-(t)}{\tau^-} + S_i(t)$ with postsynaptic spike train $S_i(t)$.<br>$\frac{d}{dt} z_i^{\text{slow}}(t) = -\frac{z_i^{\text{slow}}(t)}{\tau^{\text{slow}}} + S_i(t)$
**Online rule**	$$\frac{d}{dt} w_{ij} = S_i(t) \left(A z_j^+ z_i^{\text{slow}}(t-\epsilon) - \beta \left(z_i^-(t-\epsilon)\right)^3 (w_{ij} - \tilde{w}_{ij}) \right) - S_j(t) \left(B z_i^-(t) - \delta \right)$$ which is constrained to $w^{\text{min}} = 0 < w_{ij} < 5 = w^{\text{max}}$.
**Consolidation**	$$\tau^{\text{cons}} \frac{d}{dt} \tilde{w}_{ij} = -\tilde{w}_{ij} + w_{ij} - P\, \tilde{w}_{ij}(t) \left(\frac{w^{\text{P}}}{2} - \tilde{w}_{ij}(t) \right) (w^{\text{P}} - \tilde{w}_{ij}(t))$$ which is integrated with step size of 1.2s.
**Meta-plasticity**	Where this is mentioned explicitly we allow B_i to have a slow time dependence[3,4]: $$B_i(t) = \begin{cases} A C_i(t) & \text{for } C_i(t) \leq 1 \\ A & \text{otherwise} \end{cases}$$ with $$\frac{d}{dt} C_i(t) = -\frac{C_i(t)}{\tau^{\text{hom}}} + \left(z_i^{\text{ht}}(t)\right)^2$$ where $\frac{d}{dt} z_i^{\text{ht}}(t) = -\frac{z_i^{\text{ht}}(t)}{\tau^{\text{ht}}} + S_i(t)$.

D4	Plasticity Model
**Name**	Inhibitory Spike Timing Dependent Plasticity (iSTDP)
**Type**	Symmetric iSTDP with a constant offset for presynaptic spikes and a global modulation
**Acts on**	IE
**Synaptic traces**	$\frac{d}{dt} z_i(t) = -\frac{z_i(t)}{\tau^{\text{iSTDP}}} + S_i(t)$
**Global modulation**	$G(t) = H(t) - \gamma$ with $\frac{d}{dt} H(t) = -\frac{H(t)}{\tau^{\text{H}}} + \sum_{i \in \text{exc}} S_i(t)$
**Online rule**	$$\frac{d}{dt} w_{ij}(t) = \eta\, G(t) \left[(z_i(t) + 1)\, S_j(t) + z_j(t)\, S_i(t) \right]$$ which is constrained to $w^{\text{min}} = 0 < w_{ij} < 5 = w^{\text{max}}$.

E	Input
**Name**	Stimulus group
**Type**	Rate coded Poisson input
**Size**	4,096 Poisson neurons
**Firing rates**	$\nu_i = \nu^{\mathrm{bg}} + \nu^{\mathrm{scale}}\ \xi_i(t)$
**Input pattern**	$$\xi_i(t) = \begin{cases} 0 & \text{no active input pattern} \\ \xi_i^\mu & \text{if pattern } \mu \text{ is active} \end{cases}$$ where the ξ_i^μ are 64×64 grayscale images interpreted as 1D vectors and normalized to [0,1]. Only a single pattern can be active at a time. A pattern stays active during a finite period drawn from an exponential distribution with mean T^{On}. Each pattern activation is followed by a period of inactivity with a duration drawn from another exponential distribution with mean T^{Off}.

F	Measurements
**Type**	**Description**
	Spike activity for raster and activity plots and weight matrices at the end of the simulation.

Supplementary Table 2 – Simulation parameter summary of network model.

**Populations**		
**Name**	**Value**	**Description**
N_{E}	4,096	Size of excitatory population E
N_{I}	1,024	Size of inhibitory population I
N_{Stim}	4,096	Size of external population Stim

**Connectivity**		
**Name**	**Value**	**Description**
ϵ	0.1	Probability of connection (EE,EI,IE,II)
ϵ^{Stim}	0.05	Probability of connection (StimE)
$\tilde{w}$	0.0	Initial reference weight for all connections
w^{EE}	0.1	Initial excitatory weight
w^{EI}	0.2	Excitatory weight
w^{IE}	0.15	Initial inhibitory weight
w^{II}	0.2	Inhibitory weight
w^{StimE}	0.5	Initial excitatory weight

**Neuron Model**		
**Name**	**Value**	**Description**
τ	20 ms	Membrane time constant
U^{rest}	−60 mV	Resting potential
U^{exc}	0 mV	Excitatory reversal potential
U^{inh}	−80 mV	Inhibitory reversal potential
α^{E}	0.2	AMPA/NMDA ratio (excitatory population)
α^{I}	0.3	AMPA/NMDA ratio (inhibitory population)
τ^{ampa}	5ms	AMPA decay time constant
τ^{gaba}	10ms	GABA decay time constant
τ^{nmda}	100ms	NMDA decay time constant
τ^{a}	100ms	Adaptation time constant
Δ^{a}	0.1	Adaptation strength
τ^{b}	20s	Slow adaptation time constant
Δ^{b}	5×10^{-4}	Slow adaptation strength
τ^{thr}	2ms	Threshold time constant
$\vartheta^{\mathrm{rest}}$	–50mV	Threshold resting value

Short term plasticity model (excitatory synapses, D2)		
**Name**	**Value**	**Description**
τ^{d}	200ms	Depression time constant
τ^{f}	600ms	Facilitation time constant
U	0.2	Initial release probability parameter

Plasticity Model (excitatory synapses, D3)		
**Name**	**Value**	**Description**
τ^{+}	20ms	Presynaptic trace for excitatory plasticity
τ^{-}	20ms	Postsynaptic trace for excitatory plasticity
τ^{slow}	100ms	Slow postsynaptic trace for excitatory plasticity
A	1×10^{-3}	LTP rate
B	1×10^{-3}	LTD rate (in simulations without metaplasticity)
δ	2×10^{-5}	Transmitter triggered plasticity strength
β	0.05	Heterosynaptic plasticity strength parameter
τ^{cons}	20min	Consolidation time constant
w^{min}	0	Minimum weight value
w^{max}	5	Maximum weight value
w^{P}	0.5	Upper fixed point of reference weight potential
P	20	Potential strength parameter
τ^{hom}	20min	Metaplasticity time constant (only designated simulations with metaplasticity)
τ^{ht}	100ms	Activity trace time constant for metaplasticity

Plasticity Model (inhibitory synapses, D4)		
**Name**	**Value**	**Description**
τ^{iSTDP}	20ms	STDP trace time constant
γ	4Hz	Target population rate
τ^{H}	10s	Global secreted factor time constant
η	2×10^{-5}	Learning rate

Stimulus Model		
**Name**	**Value**	**Description**
ν^{bg}	10Hz	Background firing rate
ν^{scale}	35Hz	Maximum rate increase
T^{On}	1s	Mean stimulus-on period
T^{Off} $t < 1\mathrm{h}$	2s	Mean stimulus-off period
T^{Off} $t \geq 1\mathrm{h}$	20s	Mean stimulus-off period (unless stated otherwise)

Supplementary Methods

Population rate model. To better understand the stability properties of our learning rules, we studied a population rate model. In particular, we explicitly modeled one inhibitory population and two excitatory populations which are characterized by their firing rates ν_{inh}, ν_{a} and ν_{b}, respectively. The two excitatory populations correspond to an "assembly" population with firing rate ν_{a} and a "background" population (ν_{b}). All three populations interact with each other via synaptic couplings w_{xy}, each of which has to be understood as the combined effect of many individual synaptic connections which "see" similar pre- and postsynaptic activity. A schematic representation of the model is given in Supplementary Fig. 1 e.

We modeled the steady state response of a neuronal population to an input h

$$g(h) = \begin{cases} \nu^{\max} & \text{for } \exp(h-5) > \nu^{\max} \\ \exp(h-5) & \text{otherwise} \end{cases} \tag{1}$$

where the maximum firing rate $\nu^{\max}$ was set to 300 Hz (see inset, Supplementary Fig. 1 a). Each neuronal population followed the external input h on the rapid timescale of population dynamics $\tau_{\mathrm{pop}}^{\mathrm{a}} = \tau_{\mathrm{pop}}^{\mathrm{b}}$, which we fixed to 0.1 s ($\tau_{\mathrm{pop}}^{\mathrm{inh}} = 0.05$s for the inhibitory population). The overall temporal evolution of a population with index x, where x stands for a, b, or inh can be described as follows

$$\tau_{\mathrm{pop}}^{x} \frac{d\nu_x}{dt} = -\nu_x + g\left[\gamma_{\mathrm{exc}}\left(f\, w_{x\mathrm{a}}\, s(\nu_{\mathrm{a}}) + (1-f)\, w_{x\mathrm{b}}\, s(\nu_{\mathrm{b}})\right) + \gamma_{\mathrm{ext}} w_{xe} s(\nu_{\mathrm{ext},x}) - \gamma_{\mathrm{inh}} w_{\mathrm{x}i} \nu_{\mathrm{inh}}\right] \tag{2}$$

where $f = 0.15$ characterizes the size of the putative assembly and differences in gain of the different populations were modulated by the gain factors $\gamma_{\mathrm{exc}} = 20$, $\gamma_{\mathrm{ext}} = 10$ and $\gamma_{\mathrm{inh}} = 2$. To capture the effect of short-term plasticity (STP) of excitatory connections in the rate model we introduced the steady-state STP function s based on previous work[2], defined as $s(\nu) \equiv x(\nu)u(\nu)\nu$ with

$$x(\nu) = \frac{1}{1 + \tau_{\mathrm{d}} u(\nu)\nu} \tag{3}$$

$$u(\nu) = \frac{\nu\tau_{\mathrm{f}} U_0 + U_0}{1 + \tau_{\mathrm{f}} \nu U_0} \quad . \tag{4}$$

where τ_{d}, τ_{f} and U_0 refer to the parameters of the STP model of Eqs. (9) and (10) in the main text[2] (cf. Supplementary Fig. 1 b).

Weights involving the inhibitory population were set statically to $w_{\mathrm{a,inh}} = w_{\mathrm{b,inh}} = w_{\mathrm{inh,inh}} = 0.2$ and $w_{\mathrm{inh,a}} = w_{\mathrm{inh,b}} = 0.6$ like in the main simulation. All other weights were plastic following the equivalent rate model of the orchestrated plasticity rules

$$\frac{d}{dt} w_{yx}(t) = A\tau^{+}\tau^{\mathrm{slow}}\, \nu_x \nu_y^2 \qquad \text{triplet LTP} \tag{5}$$

$$- A\tau^{-}\, \nu_x \nu_y \qquad \text{doublet LTD} \tag{6}$$

$$- \beta\, \left(w_{yx} - \tilde{w}_{yx}(t)\right) \xi(\nu_y) \qquad \text{heterosynaptic} \tag{7}$$

$$+ \delta\, \nu_x \qquad \text{transmitter-induced} \tag{8}$$

in which all parameters, if not mentioned otherwise, were identical to those in the plasticity model for the spiking neurons and $\xi(\nu)$ is the burst detector, which, in the spiking model, contains the third power of a synaptic trace multiplied by the postsynaptic spike train (see Eq. (13) in the main text). To convert

it to a temporal average in the rate model[4] we compute the expectation value of the third moment of this synaptic trace $\left\langle z_i^-(t)^3 \right\rangle$ assuming Poisson firing. The expression can be obtained conveniently by taking the Laplace transform of the stationary distribution of z_i^- when interpreting it as a random variable (see the derivation below) which yields $\xi(\nu_y) = \frac{1}{6}\left(2 + 9\nu_y\tau + 6\nu_y^2\tau^2\right)\nu_y^2$. Since neurons in our spiking simulation are refractory, a fact which is not accounted for in the Poisson assumption, the parameter β in Supplementary Eq. (7) was lowered to $\beta = 0.0065$ in the rate model. Finally, the consolidation dynamics behind $\tilde{w}$ were the same as in the spiking model (Supplementary Table 2), with a minor parameter change to $P = 25$.
To generate the plots in the Supplementary Figs. 1 and 2, the 15 state variables (three rates, 6 weights and 6 reference weights) were integrated using Python code with the SciPy module. External input to both populations was constant at 10Hz except during external stimulation. To simulate the stimuli shown in Supplementary Fig. 2 a we transiently increased the external input rate to the assembly population $\nu_{\mathrm{ext},x}$. Specifically we added to the spontaneous firing rate of 10Hz a Gaussian temporal profile with a standard deviation of 0.5 s and an amplitude of 80 Hz (Supplementary Fig. 2 a) and 15Hz for recall and -10Hz (resulting at 0Hz total firing rate) to terminate a recall state (Supplementary Fig. 2 b).

Derivation of the moments of a synaptic trace for Poisson firing statistics. We can describe the probability flux of a synaptic trace z defined by the ordinary differential equation

$$\frac{dz}{dt} = -\frac{z}{\tau} + aS(t) \tag{9}$$

with time constant τ and spike train $S(t)$ by the following partial differential equation

$$\begin{aligned} \partial_t p(z,t) &= \frac{1}{\tau}\partial_z(zp(z,t)) - \lambda p(z,t) + \lambda p(z-a,t) && (10) \\ &= \frac{1}{\tau}p(z,t) + \frac{z}{\tau}\partial_z p(z,t) - \lambda p(z,t) + \lambda p(z-a,t) && (11) \end{aligned}$$

where the terms involving λ describe the sink and source terms that capture the jumps of size a caused by spikes at rate λ.
We now require stationarity $\partial_t p = 0$, and find

$$z\frac{d}{dz}p(z) = -p(z) + \lambda\tau\, p(z) - \lambda\tau\, p(z-a) \tag{12}$$

We now compute the Laplace transform of the left hand side of Supplementary Eq. (12):

$$\begin{aligned} \int_0^\infty dz\, e^{-sz} z\frac{dp}{dz} &= \left[ze^{-sz}p\right]_0^\infty - \int_0^\infty dz\frac{d}{dz}\left(e^{-sz}z\right)p && (13) \\ &= -\int_0^\infty dz\, p\left(e^{-sz} - sz\, e^{-sz}\right) && (14) \\ &= -\bar{p}(s) + s\int_0^\infty dz\, p\, ze^{-sz} && (15) \\ &= -\bar{p}(s) - s\frac{d}{ds}\bar{p}(s) && (16) \end{aligned}$$

which we now insert into Suppl. Eq. (12):

$$-\bar{p} - s\frac{d\bar{p}}{ds} = -\bar{p} + \lambda\tau\bar{p} - \lambda\tau\, e^{-sa}\bar{p} \tag{17}$$

$$\frac{d\bar{p}}{ds} = -\lambda\tau\frac{1-e^{-sa}}{s}\bar{p} \tag{18}$$

and therefore $\bar{p}(s) = \exp\left(-\lambda\tau \int_0^s \frac{1-e^{-sa}}{s'}ds'\right)$. The moments μ_n of z are then given by

$$\mu_n = (-1)^n \lim_{s\to 0}\frac{d^n}{ds^n}\bar{p}(s) \tag{19}$$

which yields the required third moment for the rate model above as $\frac{1}{6}\lambda\tau\left(2 + 9\lambda\tau + 6\lambda^2\tau^2\right)$. We summarize the first 5 moments for an assumed jump size of $a = 1$ in the following table:

n	$\langle z^n \rangle$
1	$\lambda\tau$
2	$\frac{1}{2}\lambda\tau\,(1 + 2\lambda\tau)$
3	$\frac{1}{6}\lambda\tau\left(2 + 9\lambda\tau + 6\lambda^2\tau^2\right)$
4	$\frac{1}{12}\lambda\tau\left(3 + 25\lambda\tau + 36\lambda^2\tau^2 + 12\lambda^3\tau^3\right)$
5	$\frac{1}{60}\lambda\tau\left(12 + 175\lambda\tau + 425\lambda^2\tau^2 + 300\lambda^3\tau^3 + 60\lambda^4\tau^4\right)$

Supplementary references

[1] Mongillo, G., Hansel, D. & van Vreeswijk, C. Bistability and spatiotemporal irregularity in neuronal networks with nonlinear synaptic transmission. *Phys Rev Lett* **108**, 158101 (2012).

[2] Mongillo, G., Barak, O. & Tsodyks, M. Synaptic theory of working memory. *Science* **319**, 1543–1546 (2008).

[3] Bienenstock, E., Cooper, L. & Munro, P. Theory for the development of neuron selectivity: orientation specificity and binocular interaction in visual cortex. *J Neurosci* **2**, 32–48 (1982).

[4] Pfister, J.-P. & Gerstner, W. Triplets of spikes in a model of spike timing-dependent plasticity. *J Neurosci* **26**, 9673–9682 (2006).
